# Evidence for the Range Expansion of Ciguatera in French Polynesia: A Revisit of the 2009 Mass-Poisoning Outbreak in Rapa Island (Australes Archipelago)

**DOI:** 10.3390/toxins12120759

**Published:** 2020-12-01

**Authors:** Mireille Chinain, Clémence Mahana iti Gatti, André Ung, Philippe Cruchet, Taina Revel, Jérôme Viallon, Manoëlla Sibat, Patrick Varney, Victoire Laurent, Philipp Hess, Hélène Taiana Darius

**Affiliations:** 1 Institut Louis Malardé, Laboratory of Marine Biotoxins—UMR EIO (IFREMER-ILM-IRD-UPF), P.O. Box 30, 98713 Papeete, Tahiti, French Polynesia; cgatti@ilm.pf (C.M.i.G.); aung@ilm.pf (A.U.); pcruchet@ilm.pf (P.C.); trevel@ilm.pf (T.R.); jviallon@ilm.pf (J.V.); tdarius@ilm.pf (H.T.D.); 2 Institut Français de Recherche Pour l’Exploitation de la Mer, Phycotoxins Laboratory, 44311 Nantes, France; manoella.sibat@ifremer.fr (M.S.); philipp.hess@ifremer.fr (P.H.); 3 Météo France, Direction Inter-Régionale en Polynésie Française, P.O. Box 6005, 98702 Faa’a, Tahiti, French Polynesia; patrick.varney@pfmail.dirpf.meteo.fr (P.V.); victoire.laurent@meteo.fr (V.L.)

**Keywords:** ciguatera poisoning, French Polynesia, *Gambierdiscus*, ciguatoxins, epidemiology, toxicological analyses, risk management, climate change

## Abstract

Ciguatera poisoning (CP) results from the consumption of seafood contaminated with ciguatoxins (CTXs). This disease is highly prevalent in French Polynesia with several well-identified hotspots. Rapa Island, the southernmost inhabited island in the country, was reportedly free of CP until 2007. This study describes the integrated approach used to investigate the etiology of a fatal mass-poisoning outbreak that occurred in Rapa in 2009. Symptoms reported in patients were evocative of ciguatera. Several *Gambierdiscus* field samples collected from benthic assemblages tested positive by the receptor binding assay (RBA). Additionally, the toxicity screening of ≈250 fish by RBA indicated ≈78% of fish could contain CTXs. The presence of CTXs in fish was confirmed by liquid chromatography tandem mass spectrometry (LC-MS/MS). The potential link between climate change and this range expansion of ciguatera to a subtropical locale of French Polynesia was also examined based on the analysis of temperature time-series data. Results are indicative of a global warming trend in Rapa area. A five-fold reduction in incidence rates was observed between 2009 and 2012, which was due in part to self-regulating behavior among individuals (avoidance of particular fish species and areas). Such observations underscore the prominent role played by community outreach in ciguatera risk management.

## 1. Introduction

Increasing numbers of poisonings linked to the consumption of seafood contaminated with marine toxins are being reported worldwide [1,2,3,4]. Among the dozen of poisoning syndromes described so far [5], ciguatera poisoning (CP) represents the most common non-bacterial seafood poisoning globally. Ciguatera results from the consumption of fish contaminated with lipid soluble toxins known as ciguatoxins (CTXs), which originate from assemblages of epiphytic dinoflagellates of the genera *Gambierdiscus* and *Fukuyoa* [6,7,8] (for reviews, and references therein). Yet, other marine organisms such as bivalves, echinoderms, gastropods, and arthropods may also be involved [9,10,11,12,13,14]. *Gambierdiscus* and *Fukuyoa* spp. algal ciguatoxins are readily transferred through the food web from algae to herbivorous and then carnivorous fish, and ultimately to humans [15,16].

Ciguatera is characterized by a complex array of gastrointestinal, neurological, and cardiovascular symptoms of variable intensity, which are often complicated by chronic manifestations that can last for months to years [17,18,19,20]. The most commonly reported symptoms include diarrhea, vomiting, paresthesia of extremities, circumoral paresthesia, cold allodynia, generalized pruritus, myalgia, arthralgia, bradycardia, hypotension, extreme fatigue, etc. [19]. Although ciguatera-related fatalities are rare (<0.1% of reported cases), the high morbidity of this debilitating and sometimes long-lasting illness makes it both a prominent public health issue [4,15,19] and a major impediment for subsistence and recreational fisheries worldwide [21,22,23,24].

Until the 2000s, the distribution of ciguatera-causing organisms and ciguatoxic fish were thought to be limited to regions between latitudes 35° N and 35° S, with the highest poisoning incidence rates consistently reported from two tropical, historical CP-endemic areas, the Pacific and Caribbean regions [4] (for review and references therein) and [25,26], which is a situation due in part to the strong reliance of local communities on marine resources. The first reports of consumer illness and/or detection of ciguatoxic fish caught in previously non-endemic areas can be found in the literature as early as 2004 from localized areas such as the Macaronesia (Canary Islands, Madeira) [27,28,29,30,31,32,33,34,35,36], the eastern Mediterranean [37], the Gulf of Mexico [38], or the coast of Cameroon in West Africa [21]. In most of these instances, confirmation of the presence of CTXs in implicated toxic meals was consistent with the detection of *Gambierdiscus* species within the respective regions [38,39,40,41,42,43,44,45,46,47,48].

The current expansion of CP to novel areas such as Macaronesia and east and southeast Asia since the 2000s is now well established [4]. Multiple factors are believed to contribute to this expansion of the geographical range of CP, such as anthropogenic disturbances [49,50,51], naturally occurring environmental changes, including long-term climatic oscillations and global warming [52,53], which likely provide favorable conditions for the migration and settlement of CP causative organisms in temperate-like (and warmer) areas of the globe [4,54] (for reviews and references therein). Modeling CP occurrences based on long-term time-series data and/or temperature projections has also been addressed in several papers [55,56]; however, few studies have actually provided a clear assessment of the link between global warming and increased number of CP cases [25,57,58,59].

**Figure 1:**
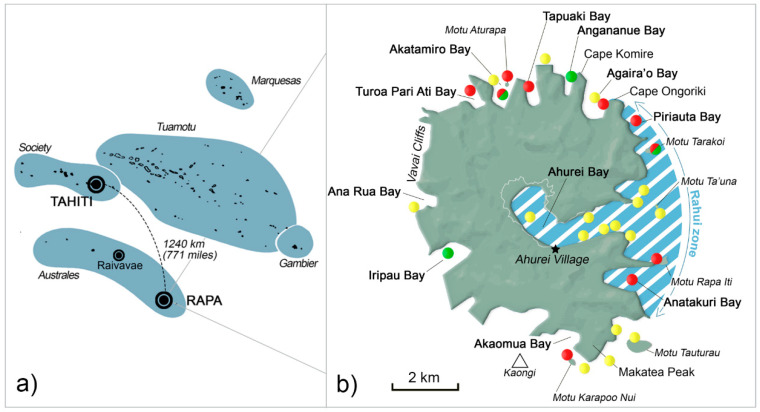
Maps of (**a**) French Polynesia and (**b**) Rapa Island (Australes archipelago). Yellow dots represent the 29 sampling sites prospected during the 2010 field study. The sampling sites highlighted in green and/or red indicate the locations where assemblages of *Gambierdiscus* spp. and/or *Ostreopsis* spp., respectively, were found on various macroalgal host species.

French Polynesia is a long-standing ciguatera hotspot as evidenced by the epidemiological data available for this country since 1960 [17,60,61,62]. Since 2007, a country-wide epidemiological surveillance program of CP cases is conducted jointly by the Institut Louis Malardé and the Public Health Directorate of French Polynesia (www.ciguatera.pf [63]). None of the five archipelagoes of French Polynesia is completely immune from ciguatera [23]. Although the general trend observed in ciguatera annual incidence rate (IR) points toward a stable IR in French Polynesia as a whole, IR rates can differ considerably from one island to another, ranging from 2 to over 1800 cases per 10,000 inhabitants [4]. As previously reported in other endemic localities of the South Pacific [62], these figures are likely under-estimated and could quite possibly be at least doubled [64]. Of note, a distance gradient is generally observed in the IRs, with the island groups furthest from Tahiti (Society archipelago) showing the highest IRs, due mainly to dietary differences observed between island groups [17]. In this respect, Rapa (Iti) Island located in the southernmost part of the Australes archipelago (Figure 1a) stood as an exception: until 2007, this island was reputed free of ciguatera [60], whereas its sister island Raivavae consistently showed some of the highest IRs in the Australes between 2007 and 2008 [50]. This small island (40 km^2^) is commonly referred to as the “little sister” of Rapa Nui (Easter Island), and it is regarded as one of the most isolated islands in the South Pacific, along with Pitcairn and Easter Island: indeed, there is no airport on Rapa Island (Figure 1b) and it takes around 50 h to reach the island by cargo ship from Tahiti. Boats traveling there are few and far between (every two to three months), and patrol boats of the French Navy also carry out episodic liaison missions from Tahiti. Due to its extreme southern location at a temperate-like latitude, Rapa’s climate is characterized by seasonal variations more pronounced than in the rest of French Polynesia, with an annual average temperature of 20 °C and an important precipitation regime [65].

In October 2009, an unprecedented mass-poisoning outbreak occurred in this island following community fishing in the *rāhui* (conservation) area. Within months, this outbreak is believed to have affected nearly half of the resident population and even resulted in two fatalities. Faced with the magnitude and severity of this outbreak, the local public health authorities mandated scientists from the Institut Louis Malardé to conduct environmental and toxicological investigations in Rapa Island in order to investigate the etiology of this mass poisoning. In parallel to the field survey, a community outreach program was also conducted among the local population, as early as January 2010, and was continued throughout 2010 and in the ensuing years. In particular, individuals were advised not to consume specific parts of the fish (head, viscera) and to avoid particular fish species and fishing areas. The importance of a systematic report of CP incidents was also emphasized.

The present paper describes the epidemiological and field investigations conducted in 2010 in Rapa, as well as the benefits that aroused from community outreach interventions among the local population. In addition to analyses by the receptor-binding assay conducted in 2010, fish samples from the event were re-analyzed with recently optimized methods (Neuro2a assay and tandem mass spectrometry). Finally, retrospective temperature data series collated from different sources were also examined to assess the current climate trend in Rapa area. Taken together, our results confirm the current expansion of ciguatera to this temperate-like locale of French Polynesia, as well as the relevance and usefulness of an integrated approach in CP risk management in newly affected hotspots.

## 2. Results

### 2.1. Epidemiology of the 2009 Outbreak in Rapa Island

While no case was reported in 2007, six isolated CP incidents occurred starting from 2008, involving a carnivorous fish, *Seriola lalandi* (king fish, or *ma’aki* in native language), and an herbivorous fish, *Kyphosus cinerascens* (highfin chub or *karamami* in native language), for three (50%) and two (33%) of these cases, respectively (Table 1).

At the peak of the 2009 outbreak, a total of 87 resident people was seen in consultation at the infirmary, giving an IR of 1805 cases per 10,000 people for 2009. Of note, the majority of poisoning cases (78%) occurred in October (28%) and November 2009 (50%). The unusual severity of this outbreak even resulted in two fatalities. However, between 2009 and 2012, an 80% reduction in the number of poisoning cases was reported. Recent data point to an IR (128 cases/10,000 people on average in the past two years) comparable to the one reported in 2008 (IR = 145 cases/10,000 people) (Table 1). According to observations made by the nurse on site, first symptoms appeared between 1 and 72 h following the ingestion of the toxic meal (Table 2), except for one female patient, aged 52, who developed symptoms within minutes after consumption of the viscera of a seagrass parrotfish (*Leptoscarus vaigiensis*). Yet, apart from this rapid onset of poisoning symptoms, this patient displayed clinical signs typical of a moderate CP. Individuals presented with digestive and neurological symptoms highly evocative of ciguatera: in 2009–2010, during the peak of the epidemic, digestive disorders (diarrhea) were recorded in 68% of affected patients, while cold allodynia (84%), paresthesia (88%), dysesthesia (79%) and itching (71%) were among the predominant neurological symptoms (Table 2).

**Figure 2:**
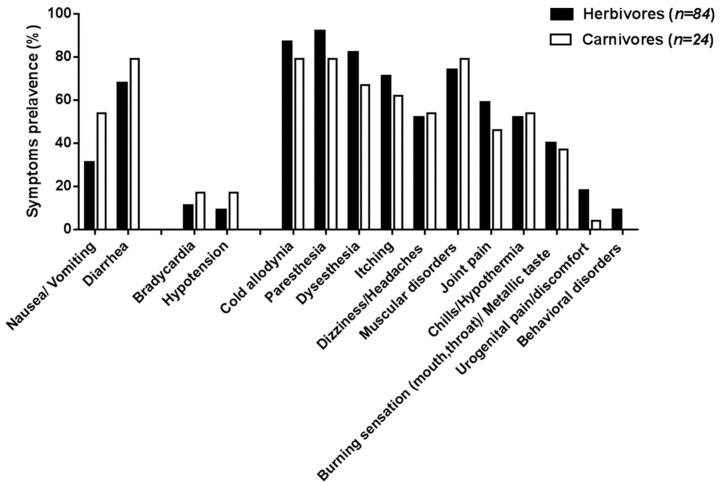
Prevalence (in percentage) of the major clinical symptoms reported in patients following the ingestion of herbivorous vs. carnivorous toxic fish during the 2009–2010 poisoning outbreak in Rapa Island. The prevalence of cardiovascular symptoms was calculated from n = 74 and n = 19 declaration forms for herbivores and carnivores, respectively.

The prevalence of symptoms reported in patients according to the trophic status of the implicated fish was also examined, showing a strong dietary preference for herbivores (n = 84) vs. carnivores (n = 24) among Rapa residents. Data in Figure 2 indicate that digestive and cardiovascular disorders were more prevalent in carnivore-based poisoning, with the caveat that this comparison is based on groups with different and limited sample size. Interestingly, behavioral disorders were reported only in toxic incidents following the consumption of herbivores (Figure 2).

Of the 87 patients seen at the Rapa infirmary in 2009, 54% declared having previously experienced at least one or multiple (two to three) CP-like poisoning events. Moreover, for 52% and 47% of them, fish meal included viscera and head, respectively, vs. 33% who limited their fish consumption to the flesh only. By 2010, the proportion of CP cases involving the consumption of viscera and head had decreased to 22% and 37%, respectively, whereas the percentage of patients who consumed only flesh had increased by almost two-fold (60%). Of note, two fatalities were recorded in 2009, while one patient required hospitalization in 2010. Moreover, based on declaration forms, the percentage of unreported cases (i.e., guests who had shared a toxic meal with reporting patients and went on developing symptoms, but for whom no declaration form was completed) was estimated at 40% and 10% in 2009 and 2010, respectively. Of note, this rate remained around 18% on average until 2014.

**Figure 3:**
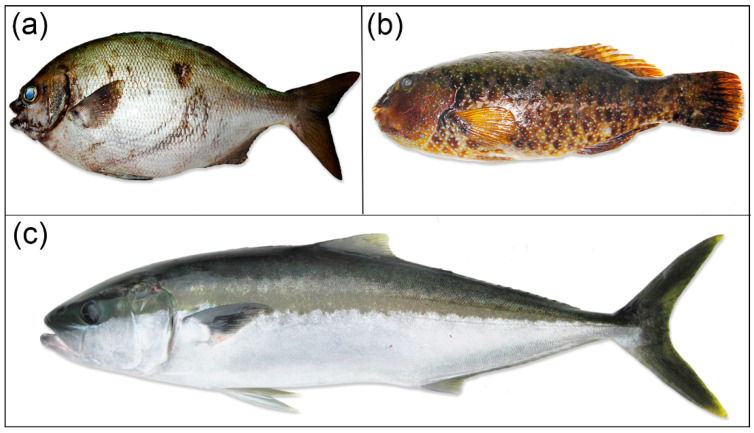
Photographs of three of the fish species regarded as major contributors to the mass-poisoning outbreak reported in Rapa Island: (**a**) *Kyphosus cinerascens* (highfin chub—karamami, herbivore); (**b**) *Leptoscarus vaigiensis* (seagrass parrotfish—komokomo, herbivore); (**c**) *Seriola lalandi* (king fish—ma’aki, carnivore). (photo credit: © Institut Louis Malardé).

Data collected at the infirmary revealed the fish involved in poisoning events were caught from two main areas, i.e., in the *rāhui* zone located in the vicinity of Ahurei village and Akatamiro Bay, a daily fishing site for local residents located north of the island, which accounted for 49 and 33% of reported events respectively, in 2009 (Figure 1 and Table 1). In addition to *S. lalandi* and *K. cinerascens*, another herbivorous species was frequently mentioned in declaration forms, namely *Leptoscarus vaigiensis* (seagrass parrotfish, or komokomo in native language) (Figure 3). All together, these three species accounted for 80 and 72% of the reported events in 2009 and 2010, respectively. Interestingly, a noticeable shift in both the fish species and fishing areas involved in CP events was observed following the 2010 field campaign and public outreach interventions in the island: indeed, this group of fish represented no more than 12, 38, and 27% of the fish involved in poisoning events in 2012, 2013, and 2014, respectively (Table 1).

Likewise, altogether, the *rāhui* zone and Akatamiro Bay, which were implicated in 82% of poisoning events in 2009, were mentioned in 41 and 28% of declaration forms in 2012 and 2013, respectively. Conversely, 35 and 44% of the implicated fishing sites in 2012 and 2013, respectively, corresponded to new fishing areas located in the north (e.g., Turoa Pari Ati Bay), south (e.g., Motu Tauturau, Akaomua Bay, Kaongi offshore shoals), or west (e.g., Iripau Bay, Ana Rua Bay, Vavai Cliffs) of Rapa Island (Figure 1 and Table 1). In 2017, no declaration form was received at all, while in 2018, 50% of the implicated fishing sites corresponded to locations other than the *rāhui* zone and Akatamiro Bay (Table 1).

### 2.2. Abundance, Distribution, and Toxicity of Gambierdiscus Populations

Overall, 50 macroalgal samples were collected in 29 distinct locations and analyzed for the presence of ciguatera-causing organisms. *Gambierdiscus* populations were present in four of the sampling sites (Figure 1 and Table 3), i.e., Motu Tarakoi, Akatamiro Bay, Cape Komire, and Iripau Bay.

**Figure 4:**
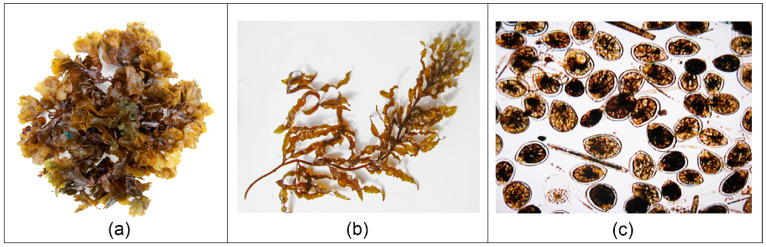
Photographs of the two dominant macroalgal hosts present in Rapa waters and wild sample of benthic microalgal species found on these substrates: (**a**) *Lobophora variegata* (Fucophyceae); (**b**) *Sargassum* sp. (Fucophyceae); (**c**) benthic assemblage composed almost exclusively of *Ostreopsis* spp cells collected from Motu Rapa Iti (photo credit: © Institut Louis Malardé).

Cell yields in *Gambierdiscus* wild samples ranged from 2250 cells (Akatamiro Bay) to 10,350 cells (Iripau Bay). *Gambierdiscus* species are not easily distinguishable by light microscopy and, unfortunately, quantitative PCR assays useful to discriminate between species of this dinoflagellate were not yet available at the time of this study. Thus, it was not possible to document the diversity and relative abundance of the different *Gambierdiscus* species present in these wild samples. *Gambierdiscus* preferred macroalgal host was *Lobophora variegata* (*kautake* in native language) (Table 3). The other dominant Fucophycea observed in Rapa waters was *Sargassum* sp. (Figure 4a,b). Interestingly, these two macroalgae were also colonized by dense populations of *Ostreopsis* spp., most notably in three sites within the *rāhui* zone where an abundance of up to 19 × 10^6^ (Piriauta Bay), 15.8 × 10^6^ (Motu Rapa Iti, Figure 4c), and 15.7 × 10^6^ cells (Cape Ongoriki) were recorded. It should be noted that an additional *Ostreopsis* spp. bloom (13 × 10^6^ cells) was also sampled from the species *Dictyota bartayresiana* (Fucophycea) collected near Motu Aturapa (Table 3).

Toxicity analyses conducted by the radioactive receptor binding assay (rRBA) confirmed a composite CTX-like activity in extracts of *Gambierdiscus* wild samples, ranging from 0.5 to 13.5 pg CTX3C eq cell^−1^ (Table 3). Of note, the sample collected from Motu Tarakoi at the peak of the poisoning outbreak (i.e., in January 2010) displayed a CTX-like activity of 1.6 pg CTX3C eq cell^−1^.

### 2.3. rRBA, CBA-N2a and LC-MS/MS Toxicity Data in Fish

A total of 251 fish specimens representing different feeding types (i.e., herbivores, carnivores, and to a lesser extent omnivores) were spear-fished and tested individually using the rRBA in the frame of a large-scale toxicity survey conducted in the same study sites as those screened for the presence of *Gambierdiscus* assemblages. Species commonly consumed in Rapa were primarily targeted, including surgeonfish, parrotfish, chubs, goatfish, groupers, and jacks (Table S1). The rRBA data allowed confirmation of a CTX-like activity in 78% of fish (Table S1), with a similar contamination rate in herbivores (85%, n = 159) vs. carnivores (78%, n = 89) (Table S1), and no significant differences between the rRBA values monitored in herbivores vs. carnivores (*p* value = 0.8109). Concerning specifically *L. vaigiensis* and *K. cinerascens*, the two herbivore fish species primarily involved in the 2009 outbreak (Table 1), rRBA data showed 92 and 79% of samples, respectively, tested rRBA positive, with maximum CTX-like activity of 12.4 and 4.1 µg CTX3C eq kg^−1^, respectively (Table S1). It should be noted that a majority of the tested fish harbored exceptionally high levels of CTX-like compounds, which are well above the concentration that causes ciguatera in humans in the Pacific, i.e., 0.1 ppb or 0.1 µg CTX1B eq kg^−1^ [15]. For example, the highest CTX-like activity found in the herbivore *L. vaigiensis* #237 and the carnivore *G. plessisi* #156 were 12.4 µg CTX3C eq kg^−1^ (or 5.2 µg CTX1B eq kg^−1^) and 14.5 µg CTX3C eq kg^−1^ (or 6.1 µg CTX1B eq kg^−1^), respectively (Table S1). No correlation was found between the size/weight of fish and rRBA values (linear correlation test, *p* values > 0.05). Interestingly, several of the study sites within the *rāhui* zone and the northern bays clearly appeared as high-risk locations for ciguatera, with >80% of positive samples. In contrast, the frequency of rRBA CTX positive fish was remarkably low in areas sampled in the south of the island, i.e., Akaomua Bay (7%, n = 15), Makatea Peak (0%, n = 4), and to a lesser extent, Ana Rua Bay (46%, n = 13) (Table S1).

Twelve fish selected among the rRBA negative (x5) and positive (x7) samples were subsequently analyzed using recently optimized protocols of the CBA-N2a [66] and liquid chromatography tandem mass spectrometry (LC-MS/MS) for confirmation of the presence of CTX congeners [67] (Table 4).

For rRBA negative samples, CBA-N2a was able to detect a low CTX-like cytotoxic activity in *C. microrhinos* #161 and *M. grandoculis* #198 (Table 4), whereas a fairly good concordance was found between rRBA and LC-MS/MS results, i.e., all negative samples by rRBA were also identified as negative by LC-MS/MS, except for *Kyphosus cinerascens* #159 in which CTXs <LOQ (limit of quantification) were detected (Table 4).

**Figure 5:**
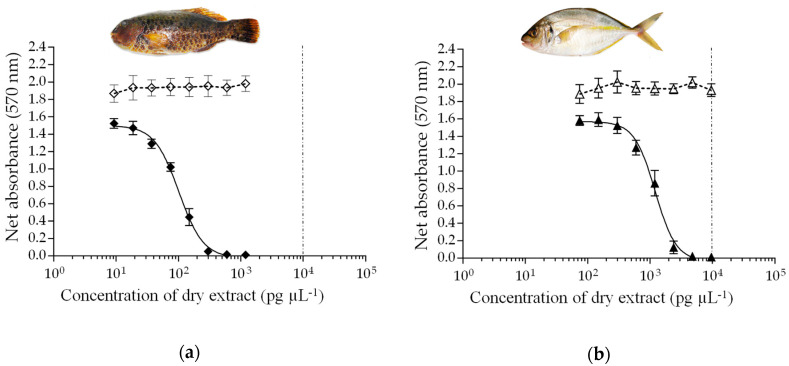
Composite cytotoxicity dose-response curves of N2a cells in OV- (open symbols) and OV+ (solid symbols) conditions when exposed to increasing concentrations of LF90/10 extracts of (**a**) *Leptoscarus vaigiensis* #214 and (**b**) *Pseudocaranx dentex* #211 collected from Rapa Island. Data represent the mean ± SD of three independent experiments, each concentration run in triplicate (n = 9). The dotted vertical line corresponds to the maximum concentration of LF90/10 dry extracts for matrix interference (MCE = 10,000 pg µL^−1^).

For rRBA positive samples, data showed the rRBA consistently gave higher estimates than CBA-N2a for five of these samples, except for *L. vaigiensis* #214 and *P. dentex* #211 for which similar CTX-like activity estimates were obtained (Table 4). When N2a cells were exposed to increasing concentrations of LF90/10 dry extracts of *L. vaigiensis* #214 (Figure 5a) and *P. dentex* #211 (Figure 5b), no cytotoxic effects were observed in the absence of ouabain (O) and veratridine (V) (i.e., OV- conditions). Conversely, a sigmoidal dose–response curve with a negative slope was obtained in OV+ conditions for both fish (Figure 5a,b). A similar pattern was observed with the LF100 dry extracts of both fish (Figure S1a,b). This response is typical of the activity of voltage gated sodium channel (VGSC) activators such as CTX3C and CTX1B (Figure S1c,d). Of note, the LF90/10 fraction concentrated 82.5 and 94.6% of the total composite CTX-like activity of *L. vaigiensis* #214 and *P. dentex* #211, respectively.

**Figure 6:**
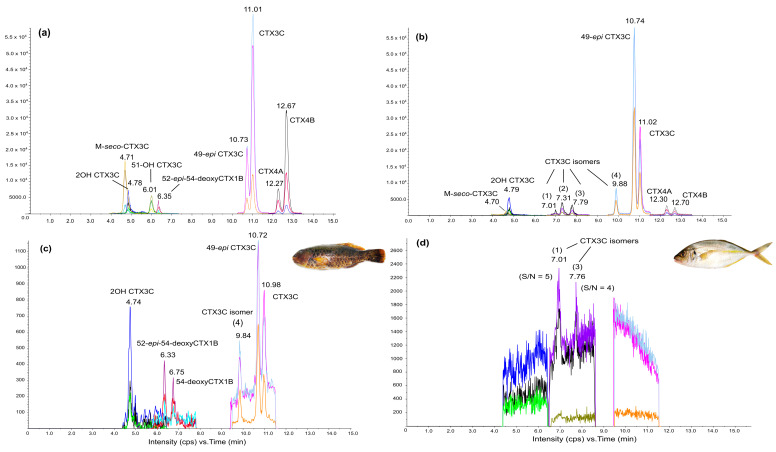
LC-MS/MS chromatograms of (**a**) mix of Pacific ciguatoxins (P-CTXs) standards (Institut Louis Malardé, Papeete, Tahiti, French Polynesia), (**b**) *Gambierdiscus polynesiensis* culture extract (NHA4, Marquesas Island), (**c**) *Leptoscarus vaigiensis* #214 fish extract, and (**d**) *Pseudocaranx dentex* #211 fish extract. The signal-to-noise (S/N) represented on chromatogram (**d**) was calculated with three standard deviations for the m/z transition 1023.6 > 1005.6.

Analyses by LC-MS/MS also confirmed the presence of CTX compounds in all positive samples, but at levels below the LOQ, except in *L. vaigiensis* #214, for which both the toxin profile and CTX estimate (0.75 µg CTX3C eq kg^−1^) were provided (Figure 6 and Table S2). Overall, six different CTX congeners could be detected in this highly toxic herbivore, i.e., in decreasing order, 49-*epi*CTX3C or CTX3B (0.24 µg CTX3C eq kg^−1^), CTX3C (0.2 µg kg^−1^), 2-hydroxyCTX3C (0.17 µg CTX3C eq kg^−1^), and a mix of 52-*epi*-54-deoxyCTX1B (or CTX2), and possibly 54-deoxyCTX1B (or CTX3) and a CTX3C isomer (4) not yet characterized at levels below the LOQ (i.e., 0.15 µg CTX3C eq kg^−1^ of fish tissue, (see Section 5.5.3) (Figure 6c). Moreover, there are some indications that the CTX-like compounds present <LOQ in other positive samples, including *P. dentex* #211, actually correspond to two additional unidentified CTX3C isomers (1) and (3) (Figure 6d). It should be noted that the three CTX3C isomers (1), (3), and (4) detected in Rapa fish are also currently found in the culture extracts of a highly toxic strain of *G. polynesiensis* (Figure 6b).

### 2.4. Temperature Trends in Rapa Island

**Figure 7:**
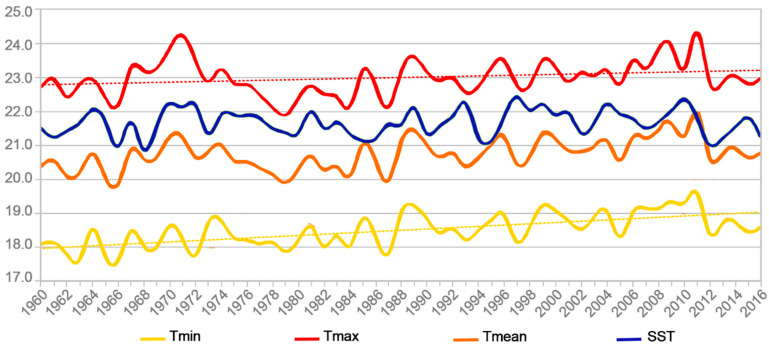
Average annual sea surface (SST) and atmospheric i.e., minimum (Tmin), maximum (Tmax), and mean (Tmean) temperatures recorded in Rapa area between 1960 and 2016. Yellow and red dotted lines represent the trend curves observed for Tmin (f(x) = 0.0188x + 17.9646) and Tmax (f(x) = 0.0085x + 22.7549), respectively.

The trends observed in the average annual minimum (Tmin), maximum (Tmax), and mean (Tmean) values of atmospheric temperature values recorded at the Rapa synoptic weather station between 1960 and 2016, were analyzed by means of the Mann–Kendall test. Results showed a significant increase of +0.86 °C in the average annual temperature values over the 57-years time period (Table 5 and Figure 7).

Of note, this increase was due more to a warming of annual minimum temperatures (+1.07 °C) than to the one of maximum temperatures (+0.48 °C) (Figure 7). Contrastingly, the positive trend (+0.48 °C) noted in the sea surface temperatures (SST) obtained from the Extended Reconstructed Sea Surface Temperature (ERSST)v5 database was not considered significant. Likewise, no significant increase was observed in the SST data provided by the Group for High Resolution Sea Surface Temperature (GHRSST) Level 4 MW_OI (data not shown).

Next, given the limited spatial resolution of ERSSTv5 (too sparse data for the Rapa area), we chose another approach by examining the potential correlations between the mean annual GHRSST, ERSSTv5, and atmospheric temperatures in Rapa. Results of the Pearson correlation test show a strong positive correlation between the GHRSST high resolution SST data and Tmin, Tmax, and Tmean for the 1998–2016 time period, both on a monthly and annual time scale (correlation coefficients r > 0.8, on average) (Table 6). In contrast, no significant correlation was found between the GHRSST and ERSSTv5 data, whatever the study period, i.e., 1998-2016 (r = 0.25) or 1960–2016 (data not shown) (Table 6).

## 3. Discussion

The present study describes the integrated approach used to investigate the etiology of an unprecedented mass-poisoning event that occurred in Rapa Island (Australes, French Polynesia) between 2009 and 2010. This approach, which combined epidemiological, environmental, and toxicological investigations, confirms this toxic outbreak as a ciguatera event and is consistent with a range expansion in the spatial distribution of both *Gambierdiscus* spp. and ciguatoxic fish to temperate-like locales of French Polynesia.

The first evidence provided is the symptomatology of the illness described in patients’ declaration forms during the peak of the outbreak. Clinically, CP is characterized by a complex combination of non-specific manifestations, with up to 175 different symptoms recorded in both the acute and chronic phases of the illness, including gastrointestinal, cardiovascular, and neurological disturbances appearing in a non-pyretic and non-allergic context [68]. In the absence of a refined, universal case definition of this illness, defining ciguatera in terms of a small, reliable group of symptoms remains difficult. However, some clues should be regarded as strong indicators of CP, especially if two or more consumers of fish at the same meal experience symptoms. Highly evocative of CP are neurological pruritus and complainants of cold allodynia, an intense and painful tingling, burning, or electric sensation in response to cold stimuli [69], which is considered characteristic of CP [17,19,70,71,72]. In the present study, almost all patients during 2009 experienced cold allodynia. The predominance of neurological symptoms over other symptoms among Rapa patients (>80% on average) is also another characteristic feature of CP illness in French Polynesia [17] and more broadly in the Pacific [19] (for a review and references therein).

Classically, ciguatera-related fatalities are rare (<0.1% of reported cases) [15,73]. In this regard, the mass-poisoning event in Rapa that resulted in two fatalities in 2009 stands out by its unusual magnitude and severity. Chan 2016 [74] has identified several contributory factors to ciguatera-related deaths, including the consumption of large portions of fish parts rich in CTXs such as viscera and head. Here, approximately half of the victims declared they had consumed the viscera and head of the fish. Yet, the limited medical resources available at the Rapa infirmary, both in terms of drug supply and supportive care capacity, may have also contributed to this high fatality rate. Finally, the potential co-exposure of Rapa Island residents to other toxin suites cannot be completely ruled out and may explain the unusual severity of the toxic incident reported in Rapa. Indeed, massive blooms of *Ostreopsis* spp were consistently observed in numerous fishing locations within the *rāhui* zone (e.g., Motu Rapa Iti, Piriauta Bay), which was incriminated in ≈50% of the reported events in 2009. *Ostreopsis* proliferation has become highly problematic in several temperate and subtropical areas due to the formation of intense blooms associated with the production of palytoxin (PLTX) and related analogs (ovatoxins, ostreocins, etc.) that have deleterious impacts on human health [54] (for review and references therein). Three distinct species, *O. lenticularis*, *O.* cf. *ovata*, and *O. siamensis*, were tentatively identified in wild samples of Rapa on the basis of their morphological characteristics [75]. These findings are consistent with previous and recent observations that these potentially toxic species frequently co-occur with *Gambierdiscus* spp. in benthic assemblages of ciguateric biotopes in French Polynesia [76,77,78,79]. Interestingly, further CBA-N2a screening of the major *Ostreopsis* spp. bloom sampled from Piriauta Bay suggested the presence of trace amounts of PLTX in this wild sample [75]. In tropical areas, PLTX-like compounds may accumulate and contaminate fish and marine invertebrates, and their ingestion by humans can have dramatic health impacts, in addition to the well-known risk of CP [80,81,82]. In Japan, Taniyama et al. (2003) [83] provided evidence that *Ostreopsis* spp. was the likely origin of PLTX bioaccumulated in the parrotfish *Scarus ovifrans* through food chain. Thus, the unusual severity of the CP toxic episode in Rapa raises the question of the potential contribution of *Ostreopsis* spp. and PLTX in the observed fatalities. Of note, LC-MS/MS analyses performed on the twelve fish specimens selected in this study failed to confirm the presence of PLTX and its derivatives in samples (data not shown).

The environmental investigations conducted in various locations around the island also confirmed the presence of *Gambierdiscus* spp. in several benthic assemblages, although these latter were often dominated by *Ostreopsis* spp. (*Ostreopsis* spp. cells were 1000-fold more abundant on average than *Gambierdiscus* spp.). Antagonist relationships between *Gambierdiscus* spp. and co-occurring *Ostreopsis* spp. have been documented in a number of field surveys [6] (for review and references therein). In vitro studies also showed that extracts of *Ostreopsis* sp. were able to suppress *Gambierdiscus* growth and adherence capacity [84,85] and that these allelopathic effects may be under the control of various environmental factors such as temperature or salinity [85]. It is hypothesized that during the time lapse between the peak of the outbreak in 2009 and samplings conducted in the frame of the present study, *Ostreopsis* spp. filled the ecological niche following *Gambierdiscus* outbreaks, as previously observed in other ciguateric locations of French Polynesia [77].

This low abundance of *Gambierdiscus* populations contrasted with the remarkably high CTX-like activities detected by rRBA in some of these samples, which were in the range of the toxic potency displayed by Pacific strains of *G. polynesiensis* as determined by either CBA-N2a or LC-MS/MS [75,86,87]. Previous field studies have outlined the frequent lack of correlation between sample toxicity and biomass [88,89] suggesting bloom toxicity and hence, the severity of CP incidents, which is primarily driven by the presence of selected, highly toxic species/strains, even if they may not be the numerically dominant ones [7,13,87]. In any case, the current knowledge gap concerning the taxonomic diversity of *Gambierdiscus* species present in Rapa waters warrants further investigations.

A total of 251 fish representative of different trophic levels were screened for their toxicity using a functional test (rRBA). This large-scale survey brought to light the high CTX-like activity measured in numerous herbivorous and carnivorous fish specimens in the Rapa food web: indeed, the highest rRBA values were measured in a herbivore (*L. vaigiensis*) and a carnivore (*G. plessisi*), and exceeded by 500 and 600 times, respectively, the advisory level of 0.01 µg CTX1B eq kg^−1^ recommended by the US Food and Drug Administration (FDA) [90], which is considered by the European Food Safety Authority (EFSA) as the concentration expected not to exert effects in sensitive individuals [91]. Long-term surveys showed there is usually a lag time of several months between the appearance of *Gambierdiscus*, the subsequent transfer of ciguatoxins into the food web, and the first reported CP cases in consumers [58,92]. The transfer of algal CTXs in the food web requires passage through herbivorous fish; therefore, in ciguatera-prone reef ecosystems, shifts in the types of reef fishes involved in CP are generally observed beginning with herbivores and then followed by carnivores years later [10,93,94]. Herein, the detection of significant amounts of ciguatoxins in a variety of fish species, including those from higher trophic levels (groupers, snappers, and jacks) suggests that ciguatera was well established in the island long before this major 2009 outbreak. The proof is, in 2009 declaration forms, an unexpected high number of patients (54%) that declared they had previously experienced one or multiple poisoning episodes, suggesting a significant number of CP cases may have been overlooked or under-reported in Rapa in months or years prior to the 2009 toxic episode. The under-reporting of ciguatera cases is one of the major causes of the underestimation in CP prevalence worldwide [4] (for a review and references therein). In the South Pacific, reported CP cases likely represent only 20% of the actual cases [62]. Based on the number of guests who shared a toxic meal with declarants and subsequently presented with symptoms, but did not report at the Rapa infirmary, it is believed that the 2009 statistics could be at least doubled.

Toxicity data from this large-scale fish survey also supported the epidemiological findings that *L. vaigiensis* and *K. cinerascens* were indeed major contributors to the mass-poisoning outbreak in Rapa in 2009, as 92% and 71% of these fish were found to be rRBA positive. Interestingly, according to the local fishermen, komokomo (*L. vaigiensis*) and karamami (*K. cinerascens*) are known to feed preferentially on *Lobophora variegata* and *Sargassum* sp., the two dominant brown macroalgal species in Rapa waters, which also appeared as *Gambierdiscus* spp. and/or *Ostreopsis* spp. preferred algal host substrates. Unlike what is observed in other CP endemic areas such as the Indian Ocean, the Caribbean, the eastern Atlantic Ocean, and the western Pacific [95,96,97,98,99], where CP events rarely involve herbivorous species, Scaridae, Acanthuridae, and Kyphosidae are frequently reported in CP cases occurring in French Polynesia and the Cook Islands [10,50,75,100]. By way of example, between 2008 and 2018, herbivorous species consistently ranked among the top five species most frequently involved in CP cases recorded annually in French Polynesia (www.ciguatera.pf [63]). Finally, analysis of the clinical data showed no striking differences in the prevalence of symptoms with regard to the trophic status of implicated fish, although the limited sample size of declaration forms prevents any definite conclusion. It is believed that the severity of CP incidents in a given area may be driven by both the genetic diversity in wild populations of the causative microalgae *Gambierdiscus* and the species-specific CTX profiles in fish, which have been shown to vary regionally [101,102]. Yet, a previous study conducted between 2002 and 2008 in French Polynesia on 3222 CP cases showed no link between the fish feeding type and the severity of CP cases (sum of symptoms) [61].

Twelve fish selected among the rRBA negative and positive fish samples were subsequently analyzed by CBA-N2a and LC-MS/MS for confirmation of the presence of CTXs. Although the limited sample size for CBA-N2a and LC-MS/MS data limits constructive comparison with rRBA results, several interesting findings were highlighted. First, for all five negative samples identified by rRBA, toxicity data were well correlated, with none of these samples containing more than trace amounts of CTX when measured by CBA-N2a (≤0.1 µg CTX3C eq kg^−1^). All positive samples by rRBA were also found positive by CBA-N2a, although rRBA consistently gave higher estimates than CBA-N2a for five of these samples, which is a result confirming previous observations in fish from the Caribbean [24,103,104,105]. This well-known tendency for overestimation by the rRBA as compared to the CBA-N2a, which has been consistently observed for samples submitted to identical extraction procedures [103,105], somewhat questions the strikingly high prevalence of toxic fish monitored in Rapa. However, from a risk management perspective, the use of rRBA as a screening tool in the present study proved highly relevant, as it likely contributed to raise immediate awareness among Rapa residents and thus limit the spread of CP. Another observed difference concerns the single fish (i.e., *L. vaigiensis* #211) in which CTXs could be detected in quantifiable amounts by LC-MS/MS, whereas rRBA and CBA-N2a were able to quantify CTX-like toxicity in all seven positive samples. This quantitation divergence between functional and chemical methods has been previously documented in large-scale field surveys conducted in the Kiribati and the Canary Islands [11,36]. It is well established that LC-MS/MS is a methodology with higher detection limits, which, additionally, can quantify only known targeted CTX analogs and may overlook congeners not yet described, while rRBA and CBA-N2a rather reflect a global response indicative of the binding affinity or cytotoxic effects of the suite of CTX compounds present in fish samples [11,36]. Additional factors may also explain these observed differences, such as the use of various extraction techniques for samples analyzed in the present study (see Section 5.4), which may have affected both the extraction efficiency and recovery of CTX compounds. Indeed, Harwood et al. (2017) [106] found that the use of methanol as the extraction solvent gives good extraction efficiency of CTXs but results in high levels of co-extractives, causing severe ion suppression with LC-MS.

The toxin profile in Rapa fish consisted of 49-*epi*CTX3C (or CTX3B), CTX3C, 2-hydroxyCTX3C, one uncharacterized CTX3C isomer, as well as two CTX analogs belonging to the CTX1B type, i.e., 52-*epi-*54-deoxyCTX1B (or CTX2) and 54-deoxyCTX1B (or CTX3), as formally identified by LC-MS/MS in the highly toxic herbivore *L. vaigiensis* #214. Two potential unidentified CTX3C isomers were also detected at levels below the LOQ in most of the positive samples identified by LC-MS/MS, including in the carnivorous fish species *P. dentex* #211. Previous field surveys have confirmed CTX3B, CTX3C, and unidentified CTX3C isomers are commonly present in food webs and marine environments of ciguateric biotopes in French Polynesia [13,14,107]. By way of example, in Anaho Bay, a long-standing CP hotspot in Nuku Hiva Island (Marquesas archipelago), a complex toxin suite comprised of CTX3B and CTX3C, 51-hydroxyCTX3C, and two congeners of the CTX1B group, i.e., CTX4A (or 52-*epi*-CTX4B) and CTX4B has been characterized in various marine invertebrates responsible for acute poisoning cases [13,14]. In the present study, the bioaccumulation of two additional CTX1B-type congeners (i.e., 52-*epi-*54-deoxyCTX1B and 54-deoxyCTX1B) was evidenced for the first time in Rapa food web, which is a finding consistent with observations in food web components of Pacific coral reef ecosystems [11]. Interestingly, several of these CTX congeners have also been formally identified in cultures of highly toxic strains of *Gambierdiscus* such as *G. polynesiensis* [87], which is a species regarded as the primary source of CTXs in French Polynesia, and the South Pacific area in general [8] (for review and references therein). These observations suggest that *Gambierdiscus* is the actual source of CTXs detected in Rapa fish, at least in herbivores. Since the genetic composition of *Gambierdiscus* populations in toxic areas may contribute in shaping the toxin profile in fish [102,108], further investigations are most needed to clarify the taxonomic diversity of *Gambierdiscus* populations in Rapa and gain insights into the toxin suites involved in local CP cases.

With regard to the climate trend status in the Rapa area, our results showed the average annual Tmin, Tmax, and Tmean values of atmospheric temperatures recorded at Rapa from 1960 to present days are trending upward, which is a strong indication of climate change in the area. However, analysis of the SST data available from both ERSST and GHRSST using the Mann–Kendall method failed to confirm this finding. This can be explained by the low spatial resolution of ERSST data that may have contributed to smooth the climate change signal and the low temporal resolution of GHRSST data. Nevertheless, the strong positive correlation between the average annual values of atmospheric temperature and GHRSST SST data for the 1998–2016 time period suggests these two datasets likely followed similar trends, thus allowing to conclude that the Rapa area is currently affected by global warming. Numerous field observations linking climate change to CP outbreaks in the tropical Pacific can be found in the literature: cyclical weather patterns such as El Niño, associated with unusual warming of Pacific Ocean waters, have resulted in spikes of ciguatera cases in the Republic of Kiribati, Western Samoa, the State of Tuvalu, and the Cook Islands [57], which is consistent with observations by Tester et al. (2010) [25] and Gingold et al. (2014) [59], who found an association between CP incidence and warmer sea surface temperatures in the Caribbean basin. While studying the fluctuations of *Gambierdiscus* populations in a ciguateric site in Tahiti Island (French Polynesia), Chinain et al. (1999) [88] found an increase in both the density and frequency of *Gambierdiscus* blooms following unusually elevated water temperatures, which is concomitant with a severe coral bleaching episode affecting large areas of the study site. As a result, the peak number of CP cases was recorded three months after these peak densities of *Gambierdiscus* [58]. Recently, Zheng et al. (2020) [56] examined the potential link between several indicators relating to SST data and ciguatera occurrences in French Polynesia and the Cook Islands, and they found that SST anomaly is proven to be a strong positive predictor of an increased ciguatera incidence for both countries. A general consensus is that global warming may lead to a substantial shift in both the distribution and abundance of ciguatera dinoflagellates [55,109], provided that species-specific habitat requirements are met [54] (for review and references therein). In addition to temperature as a potential forcing factor of CP, previous studies have also outlined reef disturbances or degradations linked to extreme climatic events (e.g., cyclone activity, heavy rains) or infestations of crown-of-thorns starfish often precede ciguatoxic events [15,23,51]. All these observations substantiate the idea that global warming as a result of climate change is among the likely causes of the observed expansion of ciguatera to Rapa Island.

However, climate may not be the only driver for ciguatera occurrence in Rapa. Local human activities resulting in significant environmental changes may also be tangible contributors to the problem, as is the case in Akatamiro Bay and Iripau Bay. Indeed, these two areas regarded as at high risk of CP (i.e., confirmed presence of *Gambierdiscus* spp. and/or *Ostreopsis* spp. in benthic assemblages, and high prevalence of toxic fish) are also the sites of major anthropogenic pressure: Akatamiro Bay is characterized by highly altered habitats, which is a likely consequence of soil erosion and subsequent runoffs (nutrient-rich inputs) from land masses due to unrestrained goat ranching, and Iripau Bay is used on a daily basis for the community-based cultivation of taro (*Colocasia esculenta*). There are some indications that nutrient levels in ciguateric biotopes correlate well with *Gambierdiscus* abundance [110,111], but this is not a consistent finding [112,113,114]. Nutrient inputs can also cause a major shift in the distribution and abundance of ciguatera-related dinoflagellates: in several locations of the Northern Great Barrier Reef (Australia) affected by ongoing coastal eutrophication, benthic assemblages in inshore reefs once dominated by *Gambierdiscus* were composed primarily of *Prorocentrum* and *Ostreopsis*, whilst *Gambierdiscus* was still dominant in offshore locations [26].

In parallel to the 2010 field survey, a community outreach program was conducted in Rapa to warn individuals against specific risk-taking behaviors and stress the importance of systematic report of CP incidents. Information about the fish species and fishing areas most at risk of ciguatera were also provided. These outreach interventions appeared to create self-regulating behavior among individuals in the years following the 2010 field campaign, as shown by (i) the noticeable shift in both fish species and fishing areas involved in CP cases; (ii) the significant drop in the prevalence of patients who declared they consumed fish viscera and/or head, and (iii) the lower under-reporting rate of CP incidents perceptible until 2014, which could also be attributed, in part, to a significant change in the population’s eating habits evidenced in the 2012–2014 declaration forms (data not shown), i.e., individuals clearly avoided sharing meals of potentially toxic fish with other (multiple) guests to limit poisoning risk. All these adjustments to CP among the local community may have contributed to the five-fold reduction in CP incidence rate between 2009 and 2012. Moreover, IRs recorded in recent years have also returned to a level comparable to 2008. It should be noted that similar results following outreach programs were achieved in Raivavae, which is another CP-prone island in the Australes archipelago [50]. In this island, educating the resident public (e.g., local leaders, fishermen, the general public, school children, etc.) about the origin of the disease and preventive measures proved useful to the community by reducing poisoning cases by two-fold. This was achieved through public meetings, dissemination of guidebooks, flyers, informational posters available in French and native language, and distributed to health centers, schools, and the town hall, as well as educational modules targeted at primary schools. All these observations support the idea that consumers’ education is critically important in the management of seafood poisonings [2].

## 4. Conclusions

The field study conducted in Rapa Island following an unprecedented toxic episode among its resident population allowed confirming that CP is no longer confined to the tropical part of French Polynesia, which is a long-standing endemic area, but should now also be considered to be well established in subtropical zones. The integrated (multi-disciplinary) approach, which combined epidemiological, environmental, and toxicological investigations, proved effective in confirming the presence of toxin-producing *Gambierdiscus* assemblages in Rapa waters. Moreover, CTX bioaccumulation in a locally caught herbivorous fish species identified as a primary contributor to the 2009 poisoning outbreak was confirmed by using three different analytical techniques. Speculation that additional toxins such as those linked to the proliferation of *Ostreopsis* spp. in the Rapa environment might have contributed to the unusual severity and magnitude of this toxic incident warrants more thorough investigations. Finally, this study also underscores the relevance and benefits of education and public outreach interventions, in parallel to field investigations, for an effective management of ciguatera risk. Expanding this sentinel approach to other ciguatera-prone areas will undoubtedly help break the persistence of this disease among French Polynesian communities.

## 5. Materials and Methods

### 5.1. Study Site

Located in the South Pacific Ocean, French Polynesia is composed of five island groups, namely Society, Marquesas, Tuamotu, Gambier, and Australes archipelagoes, spread over a surface as large as Europe (Figure 1a). Rapa Island is located in the Australes archipelago, equidistant between New Zealand and Easter Island (27.595369 S–144.362151 W) and approximately 1240 km (771 miles) south of the main island of Tahiti (Figure 1a). Monthly average maximum and minimum temperatures in Rapa range from 20.4 to 26.4 °C, and from 15.8 to 22.4 °C, respectively, with a precipitation regime of 251.7 cm of water per year on average (median value for 1981–2010) [65].

Based on the 2007 census, Rapa counted 482 inhabitants (www.ISPF.pf [115]) regrouped primarily in the main village of Ahurei (Figure 1b). The population in Rapa relies primarily on agriculture and subsistence fishing, and it is a very close-knit community. Rapa is subject to the custom of *rāhui*, which is a form of fishing taboo for conservation purposes [96,97] that restricts access to specific fishing grounds tenured by the “Conseil des Sages” of Rapa. This customary practice imposes the spatial closure of the main fishing grounds in the island during most of the year, and fish catch is allowed only at specified celebration periods.

Samples analyzed in this study were collected in the frame of a field campaign conducted in December 2010, i.e., approximately one year after the report of the mass-poisoning outbreak. A total of 29 sampling locations distributed around the island were screened for the presence of toxic *Gambierdiscus* assemblages and fish (Figure 1b).

### 5.2. Biological Samples

#### 5.2.1. Macroalgal Samples

All *Gambierdiscus* wild samples examined in the present study were collected from macroalgal substrates according to the method described in Chinain et al. (2010) [50] since the artificial substrate method (i.e., windows screens) developed by Tester et al. (2014) [116] was not yet available at the time of these field investigations. Briefly, 200 to 400 g of the most abundant and widely distributed macroalgal substrates (e.g., *Lobophora variegata* and *Sargassum* sp.) were collected at water depths between 1 and 5 m and examined for the presence of *Gambierdiscus* cells and other benthic dinoflagellate species often found in association with ciguatera-causing organisms in benthic assemblages of ciguateric biotopes, e.g., *Ostreopsis* spp. and *Prorocentrum* spp [117]. Macroalgal samples were sealed within plastic bags underwater and shaken and kneaded vigorously to dislodge dinoflagellate cells. For each macroalgal sample, the detrital suspension was successively filtered through 125, 40, and 20 μm mesh sieves. Thus, sub-samples (1 mL) of the 40 and 20 μm fractions obtained were preserved in 50 mL of 5% formalin–seawater for cell enumeration. The total cell yields were assessed under light microscopy from n = 2 to 5 counts of 100 μL aliquots. The remaining 40 and 20 µm fractions were centrifuged at 2000× *g* for 20 min, and the resulting cell pellets stored at −20 °C until further extraction.

#### 5.2.2. Fish Samples

The fish species primarily targeted in this study were those deemed edible by the local population and those most frequently involved in poisoning cases based on the ciguatera declaration forms available at the infirmary of Rapa (the only medical structure on the island) and on a questionnaire survey conducted prior to field samplings, respectively. As fish toxicity is likely to vary depending on age, several individuals per species were spear-fished to screen the largest range of size/weight possible: for instance, for *Kyphosus cinerascens*, *Leptoscarus vaigiensis,* and *Chlorurus microrhinos* catches, sample weights ranged from 235 to 3450 g, from 290 to 1070 g, and from 465 to 2300 g, respectively (Table S1). Each fish was measured and weighed, and its flesh conditioned in the form of fillets stored at −20 °C. Back at the laboratory, pooled fillets obtained from each fish specimen were thawed and homogenized by grinding in a blender (Groupe SEB, Lourdes, France) waste disposal unit and stored at −20 °C until extraction. In addition, for each fish specimen, 50 g of ground flesh tissue were transferred into a ziplock plastic bag, and freeze-dried for 20 h, at −20 °C, 1 mbar, then for 4 h, at −60 °C, 0.01 mbar (Martin Christ, Beta 1-8 LDplus) prior to LC-MS/MS analyses.

### 5.3. Epidemiological Data

The epidemiological data presented herein were compiled from patients’ declaration forms filled out by the medical staff of Rapa infirmary between 2008 and 2019. These data were collected in the frame of the country-wide epidemiological survey on CP cases currently in place in French Polynesia [4] (www.ciguatera.pf [63]). Information is gathered on age, gender, clinical symptoms reported following fish intake, number of previous poisonings, number of affected guests, as well as details of the toxic meal, e.g., fishing area, marine species involved, parts of the fish eaten, etc. (see Supplementary Material Figure S2).

### 5.4. Toxin Extraction

#### 5.4.1. Micro-Algal Samples

*Gambierdiscus* sample extracts were prepared following the protocols described in Chinain et al. (2010) [50]. Briefly, each cell pellet was extracted twice in pure methanol (MeOH) and twice in MeOH/H_2_O (50/50) under sonication for 30 min. After centrifugation, the resulting supernatants were pooled and dried under vacuum. The crude extract thus obtained was further partitioned between dichloromethane (CH_2_Cl_2_) and MeOH/H_2_O (60/40). The resulting CH_2_Cl_2_ phase, in which CTXs-like compounds are preferentially recovered, was dried under vacuum. Then, dry extracts of *Gambierdiscus* wild samples corresponding to 2250 to 10,350 cells eq (Table 3) were resuspended in 1 mL of rRBA incubation buffer [118] and stored at +4 °C until tested for their composite binding affinity via the rRBA.

#### 5.4.2. Fish Samples

*For rRBA analyses*: The extraction protocol used in 2010 followed the one described in Darius et al. (2007) [100], with slight modifications. For each fish sample, a portion of 10 g of flesh was extracted in 14 mL MeOH under sonication for 2 h and left to stand at −20 °C overnight. Then, the resulting crude extract was centrifuged and purified by solid phase extraction (SPE) technique. The supernatant from each tube was adjusted to MeOH/H_2_O (70/30) and passed through Sep-Pak C18 cartridges (360 mg; Waters^®^, Saint-Quentin, France) pre-conditioned with MeOH/H_2_O (70/30) before loading extracts. Then, after an initial washing step with MeOH/H_2_O (70/30), each column was eluted successively with MeOH/H_2_O (90/10) and pure MeOH. The resulting liposoluble fraction likely to contain the majority of CTXs (LF90/10) was further dried under vacuum, resuspended in rRBA incubation buffer at a concentration of 10 g flesh equivalent (eq) mL^−1^, and stored at +4 °C until tested for its composite binding affinity. Due to the high lipid nature of the flesh of herbivorous fish from Rapa, the precipitation (delipidation) step at −20 °C was repeated followed by a washing step using MeOH/H_2_O (80/20) prior to the elution step with MeOH/H_2_O (90/10).

*For CBA-N2a analyses*: Samples intended for CBA-N2a analyses were extracted using an optimized protocol described in Darius et al. (2018) [13]. Briefly, each sample (10 g) was extracted twice in methanol and twice in MeOH/H_2_O (50/50), under sonication for 4 h, followed by a liquid/liquid partition between CH_2_Cl_2_ and MeOH/H_2_O (60/40). The CH_2_Cl_2_ phase was dried under vacuum and defatted by a second solvent partition using cyclohexane and MeOH/H_2_O (80/20). Then, the aqueous methanol fraction was evaporated and purified on Sep-Pak C18 cartridges as previously described [13]. Then, the resulting liposoluble fractions likely to contain CTXs (i.e., LF90/10 and LF100) were dried in a SpeedVac concentrator (ThermoFisherScientific, Waltham, MA, USA) and weighed on a microbalance (model MC 410 S, Sartorius, Göttingen, Germany) at a reading accuracy of 0.1 mg. Finally, each dry extract was resuspended in MeOH at a concentration of 10 mg mL^−1^ and stored at −20 °C until tested for composite cytotoxicity.

*For LC-MS/MS analyses*: Fish extracts were prepared according to the protocols described in Sibat et al. (2018) [67], with slight modifications. Briefly, for each fish sample, freeze-dried samples corresponding to 50 g (wet weight) of flesh tissue were extracted twice with MeOH/H_2_O (90/10) (150 mL). After centrifugation at 3500× *g* for 10 min, the supernatants were pooled and evaporated at 60 °C with a rotary evaporator under reduced pressure. The residue was resuspended in MeOH/H_2_O (90/10) (30 mL) and defatted twice with *n*-hexane (60 mL). The aqueous MeOH layer was concentrated to dryness under nitrogen (N2) flux at 40 °C. The resulting crude extract was dissolved in 5 mL of ethyl acetate (EtOAc)/MeOH (85/15 v/v) prior to purification using two successive SPE clean-up steps. After conditioning with 3 mL of EtOAc/MeOH (85/15 v/v), sample extracts (2 mL, equivalent to 20 g of fish flesh wet weight) were loaded onto Bond Elut Florisil^®^ cartridges (500mg, Agilent technologies, Santa Clara, CA, USA). Cartridges were eluted with 2 × 2 mL of the same solvent. The three fractions were combined (6 mL) and evaporated under N2 flux at 40 °C. The resulting residue (E1) was dissolved in MeOH/H_2_O (70/30) (2 mL). For the second SPE purification step, Bond Elut LRC C18 cartridges (500 mg, Agilent technologies, Santa Clara, CA, USA) were used. The cartridges were first conditioned with MeOH/H_2_O (70/30) (3 mL), prior to the loading of the purified extract. Then, the C18 cartridges were washed with MeOH/H_2_O (75/25) (3 mL) and the P-CTXs were eluted with MeOH/H_2_O (90/10) (2 × 3 mL). The two eluting fractions were combined and evaporated under N2 flux at 40 °C. The resulting purified extract (E2) was resuspended in 500 µL of pure MeOH prior to LC-MS/MS analysis.

### 5.5. Toxicological Analyses

#### 5.5.1. Radioactive Receptor Binding Assay (rRBA)

*Gambierdiscus* and fish samples were initially tested for their CTX binding affinity via the radioactive receptor binding assay (rRBA) performed in a tube format following the method previously described by Darius et al. (2007) [100]. For wild *Gambierdiscus* samples, a full curve was run based on eight concentrations ranging from 2 to 900 and 12 to 4140 cell mL^−1^ for the lowest to highest cell amounts available and tested in one rRBA experiment. For fish samples, four to eight concentrations were tested in duplicate in one rRBA experiment. Radioactivity (counts per minute, cpm) was determined using a Microbeta Trilux 1450 liquid scintillation counter (Perkin Elmer, Courtaboeuf, France). The cpm data were fitted to a sigmoidal dose–response curve based on the four parameters model (4PL) using GraphPad Prism v8.4.3 software (GraphPad, San Diego, CA, USA) allowing the calculation of IC_50_ values (concentrations causing 50% inhibition of [^3^H]PbTx-3 binding on rat brain synaptosomes) expressed in cells mL^−1^ or flesh equivalent mg mL^−1^ for *Gambierdiscus* and fish samples, respectively. Calibration of the assay was achieved using CTX3C and CTX1B toxin standard solutions sourced from the Institut Louis Malardé (Papeete, French Polynesia). The CTX-like toxicity in *Gambierdiscus* and fish samples was estimated based on the comparison of their IC_50_ values with the one of CTX3C standard (IC_50_ of CTX3C/IC_50_ of sample). The CTX-like toxicity was expressed in pg CTX3C eq cell^−1^ and µg CTX3C eq kg^−1^ of fish flesh for *Gambierdiscus* and fish samples, respectively. The mean IC_50_ values obtained for CTX3C and CTX1B were 0.62 ± 0.16 and 0.26 ± 0.14 ng mL^−1^, respectively [100]; hence, to obtain rRBA values expressed in µg CTX1B eq kg^−1^, a conversion factor of x0.42 should be applied. The limit of detection (LOD) and limit of quantification (LOQ) of the assay were established at 0.015 and 0.31 pg CTX3C eq cell^−1^ for *Gambierdiscus* and 0.16 and 0.31 µg CTX3C eq kg^−1^ for fish samples. All fish samples <LOD were identified as negative samples.

#### 5.5.2. Neuroblastoma Cell-Based Assay (CBA-N2a)

Several negative and positive herbivorous and carnivorous fish specimens identified by rRBA were selected among the species primarily involved in CP incidents and/or preferentially consumed by the local population and subsequently analyzed by the neuroblastoma cell-based cytotoxicity assay (CBA-N2a) using the recently optimized protocol detailed in Viallon et al. (2020) [66]. The maximum concentrations of dry extract (MCE) that did not induce unspecific cytotoxic effects in neuroblastoma (N2a) cells were established at 10,000 and 50,000 pg µL^−1^ for F90/10 and LF100 fish extracts, respectively. First, a qualitative screening of both fractions was performed using the MCE in OV- and OV+ conditions, i.e., non-destructive Ouabain (O) and Veratridine (V) treatments between 80/8 and 90/9 µM (final concentrations) [66]. For positive fractions, the first dilution was adjusted, and a serial dilution 1:2 (eight concentrations) was undertaken to obtain a full dose–response curve run in parallel with CTX3C as the toxin standard. Another standard, namely CTX1B, was also run in parallel with the two positive samples showing the highest CTX-like activity (Figure S1c,d). Each positive fraction was tested in three independent experiments, and each concentration was run in triplicate (n = 9). Net absorbance data were fitted to a sigmoidal dose–response curve based on the 4PL model allowing the calculation of half-maximal effective concentration (EC_50_) values using GraphPad Prism v8.4.3 software. Following the determination of the EC_50_ values of CTX3C standard (EC_50_ of CTX3C, fg µL^−1^) and of the tested fraction (EC_50_ of dry extract, pg µL^−1^), the CTX-like toxicity in the fish sample (T) was estimated according to Viallon et al. 2020 [66], using the following equation:

(T) = [(EC_50_ of CTX3C)/(EC_50_ of dry extract)]*(DEW/FW),

in which T (µg CTX3C eq kg^−1^ of fish flesh) is the CTX-like toxicity, and DEW and FW represent the dry extract weight (mg) and the sample fresh weight (g), respectively. Of note, the (T) values for each fish sample indicated in Table 4 corresponded to the CTX-like activity measured in both LF90/10 and LF100 dry extracts. The LOD and LOQ of the assay for the LF90/10 fraction, which concentrates the majority of the cytotoxic activity, were determined at 0.03 ± 0.01 and 0.06 ± 0.02 µg CTX3C eq kg^−1^, respectively [66].

#### 5.5.3. Liquid Chromatography Tandem Mass Spectrometry (LC-MS/MS)

LC-MS/MS analyses were also performed on the previously selected fish samples in order to provide unequivocal confirmation of the presence of P-CTXs congeners in toxic fish and gain information on their specific toxin profiles. These analyses were conducted using a UHPLC system (UFLC Nexera, SHIMADZU, Kyoto, Japan) coupled to a hybrid triple quadrupole-linear ion-trap API4000 QTRAP mass spectrometer (SCIEX, Redwood City, CA, USA) equipped with a TurboV^®^ electrospray ionization source (ESI). A 1.8 µm C18 Zorbax Eclipse plus column (50*2.1 mm, AGILENT TECHNOLOGIES, Santa Clara, CA, USA) was employed at 40 °C and eluted at 400 µL/min with a linear gradient. Eluent A is water and eluent B is methanol, both eluents containing 2 mM ammonium formiate and 50 mM formic acid. The elution gradient ran from 78 to 88% over 10 min and was held for 4 min before re-equilibration during 5 min.

Mass spectrometry detection was operated in positive Multiple Reaction Monitoring (MRM) mode. The MRM acquisition method was created using the scheduled MRM algorithm. This algorithm optimizes the dwell times and cycle time to provide a better peak detection and improve reproducibility. A detection window of 120 s and a target scan time of 2 s were chosen for the MRM method. The selected *m/z* transitions are repertories in Table S2. The MRM experiments were established by using the following source settings: curtain gas set at 25, ion spray at 5500 V, a turbogas temperature of 300 °C, gas 1 set at 40 and gas 2 set at 60 psi, with an entrance potential of 10 V. The instrument control, data processing, and analysis were conducted using Analyst software 1.6.3 (SCIEX, Redwood City, CA, USA).

A calibration solution of CTX3C (Institut Louis Malardé, Papeete, Tahiti, French Polynesia) was prepared in MeOH with concentration ranging from 10 to 500 ng mL^−1^. The LOD and LOQ were respectively determined at 0.05 and 0.15 µg CTX3C eq kg^−1^of fish tissue, respectively. To complete chromatogram profile, a mix of Pacific CTX standards (Institut Louis Malardé, Papeete, Tahiti, French Polynesia) was injected in the sequence.

In order to quantify the P-CTX congeners and due to the lack of standards, concentrations were estimated from the P-CTX3C calibration curve assuming equivalent molar response. Thus, the respective CTX concentrations were expressed in µg CTX3C eq kg^−1^ of fish tissue.

### 5.6. Time-Series Temperature Data

Classically, 50-year temperature datasets are required to assess global warming trends, as at this timescale, low-frequency oscillations in sea surface temperature (SST) such as the Inter decadal Pacific Oscillation (IPO) interfere little with the climate change signal [119]. In the present study, both atmospheric temperature (AMT) and SST data were used to analyze the current climate change state in the Rapa area. The AMT data series for the 1960–2016 time period were obtained from the Météo France synoptic weather station located in Ahurei village in Rapa, and they were used to calculate the daily minimum (Tmin), maximum (Tmax), and mean (Tmean) values of atmospheric temperatures, as well as the average annual Tmin, Tmax, and Tmean values. The mean annual SST values were estimated from global monthly SST data available from the Extended Reconstructed Sea Surface Temperature version 5 (ERSSTv5) database that covers a time period from 1854 to present days. ERSSTv5 combines both data from in situ observations by ships and buoys and a decade of near-surface data from Argo floats [120]. For the present study, we focused on the 1960–2016 time-period using a 2° × 2° grid resolution. As a result of the poor spatial resolution of the ERSSTv5 dataset particularly in the Southern Hemisphere, we also utilized daily SST data obtained from the Group for High Resolution Sea Surface Temperature (GHRSST Level 4 MW_OI) available only from 1998/01/01 to 2017/12/31, but with a spatial resolution of 0.25° × 0.25°, which is best adapted to the scale of Rapa Island. Then, the average monthly and annual temperature data thus obtained were used to assess the potential correlations between GHRSST, ERSSTv5, and Météo France station data series, and qualify the global warming trend in the Rapa area.

### 5.7. Statistical Analyses

To determine whether rRBA values differed significantly between the herbivorous and carnivorous fish, standard deviations (SD) and 95% confidence interval (CI; *p* value < 0.05) were first calculated, and a Mann–Whitney test (non-parametric test) was performed using GraphPad Prism v8.4.3. To analyze whether there was a significant correlation between the size/weight of fish and rRBA values, a linear correlation test was performed using RStudio v1.0.153 (RStudio, Inc., Boston, MA, USA).

Climatic trend in Rapa was assessed using the Mann–Kendall test at a 95% confidence interval. Analysis was completed by estimating the linear trend with the least squares method. Given the limited sample size of the GHRSST data set (i.e., time coverage < 50 years), only the correlations between GHRSST, ERSSTv5 and Météo France data series were tested using the Pearson correlation test (CI = 0.95).

## Figures and Tables

**Table 1:** Number of ciguatera poisoning (CP) events, CP cases, and corresponding incidence rates (IR) reported from the medical facility of Rapa Island between 2007 and 2019. The prevalence (in %) of fish species and fishing sites involved in CP events is also given.

Year	2007	2008	2009	2010	2011	2012	2013	2014	2015	2016	2017 ^1^	2018	2019
**Number of CP events**		6	71	21	11	17	32	11	9	6	-	4	6
**Number of reported CP cases**	-	7	87	27	13	17	33	11	15	7	-	6	7
**Number of unreported cases** ^2^	-	5	59	3	6	2	1	-	7	5	-	1	6
**Number of fatal cases**	-	-	2 ^3^	-	-	-	-	-	-	-	-	-	-
**Incidence rates (IR)**^4^ (reported cases/10,000 inhab.)	-	145	1805	560	269	330	660	214	291	136	-	118	138
**Fish species involved (% of event)**													
*Seriola lalandi*	-	50	6	24	18	-	7	-	44	17	-	25	33
*Kyphosus cinerascens*	-	33	29	29	27	6	3	-	-	33	-	-	17
*Leptoscarus vaigiensis*	-	-	45	19	9	6	28	27	-	-	-	-	17
Others:	-	17	20	28	46	88	62	73	56	50	-	75	33
*Scombridae*		*17* ^5^	*3*										
*Carangidae*				*9*	*9*	*17*	*3*	*9*		*17*		*25*	
*Acanthuridae*						*17*			*11*				
*Scaridae*			*1*	*9*		*12*	*19*	*27*	*34*	*17*			*17*
*Serranidae*			*3*		*9*	*12*	*34*	*18*	*11*			*50*	*16*
*miscellaneous*			*13*	*10*	*28*	*30*	*6*	*19*		*16*			
**Fishing sites involved (% of event)**													
*Rāhui* zone	-	67	49	33	45	35	12	18	11	67	-	25	17
Akatamiro Bay	-	-	33	-	-	6	16	9	-	-	-	-	17
Others:	-	-	14	53	27	35	44	18	67	-	-	50	33
*Turoa Pari Ati Bay*			*2*	*10*									
*Tapuaki Bay*			*4*	*5*									
*Agaira’o Bay*							*10*						
*Kaongi*				*5*									
*Motu Tauturau*							*10*		*11*				
*Akaomua Bay*				*5*			*3*						
*Iripau Bay*				*14*			*6*	*9*	*11*				
*Ana Rua Bay*				*5*	*9*			*9*				*25*	
*Vavai Cliffs*			*1*		*9*	*23*	*3*					*25*	
*offshore*			*7*	*9*	*9*	*12*	*12*		*45*				*33*
Unknown	-	33	4	14	28	24	28	55	22	33		25	33

^1^ No data available for 2017. ^2^ Number of guests who shared a toxic meal with reporting patients and developed poisoning symptoms, but for whom no declaration form was completed. ^3^ Fatal cases involved an elderly woman with known comorbidity factors and a middle-aged man, who both presented pronounced cardiovascular symptoms. ^4^ IRs were calculated according to the population data from the 2007, 2012, and 2017 census. ^5^ Numbers in italics give the respective prevalence of fish families and fishing sites involved in CP events totalized under the “Others” headings.

**Table 2:** Clinical signs experienced by patients during the 2009–2010 mass ciguatera outbreak in Rapa Island. The number of patients concerned by each clinical sign and corresponding percentage (%) is given. Symptoms most frequently recorded are highlighted in bold characters.

Year	2009	2010
**Total number of CP cases reported**	87	27
**Digestive disorders**		
Nausea/vomiting	28 (32%)	11 (39%)
**Diarrhea**	**59 (68%)**	19 (68%)
**Cardiovascular disorders ^1^**		
Bradycardia	10 (14%) ^1^	6 (22%)
Hypotension	9 (12%) ^1^	3 (11%)
**Neurological and systemic disorders**		
**Cold allodynia**	**80 (92%)**	15 (54%)
**Paresthesia**	**83 (95%)**	17 (61%)
**Dysesthesia**	**75 (86%)**	15 (54%)
**Itching**	**69 (79%)**	12 (43%)
Dizziness/headaches	50 (57%)	10 (36%)
**Muscular disorders**	**67 (77%)**	19 (68%)
**Joint pains**	**56 (64%)**	11 (39%)
**Chills/hypothermia**	**54 (62%)**	8 (29%)
Burning sensation (throat, mouth)/”metallic” taste	43 (49%)	4 (14%)
Urogenital burning/pain/discomfort	15 (17%)	4 (14%)
Behavioral disorders (agitation, disorientation)	9 (10%)	2 (7%)

^1^ For 2009, the prevalence of cardiovascular symptoms was estimated from n = 72 declaration forms (data not available for 15 patients). Data source: Bureau de Veille Sanitaire, Public Health Directorate of French Polynesia, and Institut Louis Malardé.

**Table 3:** *Gambierdiscus* spp. and *Ostreopsis* spp. cell abundance in benthic assemblages found on various macroalgal host species. The ciguatoxins (CTX)-like activity data for *Gambierdiscus* wild samples as assessed by the radioactive receptor binding assay (rRBA) are also presented.

Genus	*Gambierdiscus* spp.			*Ostreopsis* spp.	
Sampling Sites	Macroalgal Host Species	Abundance (Cells)	CTX-Like Activity ^2^	Macroalgal Host Species	Abundance (Cells)
Turoa Pari Ati Bay				*Lobophora variegata*	6.1 × 10^6^
Akatamiro Bay	*Lobophora variegata*	2250	13.5	*Lobophora variegata*	5.4 × 10^6^
Motu Aturapa				*Dictyota bartayresiana*	13 × 10^6^
Cape Komire	*Dictyota dichotoma*	10,300	0.5		
Cape Ongoriki				*Sargassum* sp.	15.7 × 10^6^
Piriauta Bay ^1^				*Sargassum* sp.	19 × 10^6^
Motu Tarakoi ^1^	*Lobophora variegata*	5000	1.6 *	*Sargassum* sp.	3.4 × 10^6^
Motu Rapa Iti ^1^				*Lobophora variegata*	15.8 × 10^6^
Anatakuri Bay ^1^				*Lobophora variegata*	2.2 × 10^6^
Motu Karapoo Nui				*Lobophora variegata*	1.7 × 10^6^
Iripau Bay	*Lobophora variegata*	10,350	3.5		

^1^*Rāhui* zone. ^2^ Composite CTX-like activity expressed in pg CTX3C eq cell^−1^ (n = 1). * Wild sample collected in January 2010 during the peak of the outbreak.

**Table 4:** CTX-like activity estimates (expressed in µg CTX3C eq kg^−1^) as assessed by rRBA, CBA-N2a, and LC-MS/MS in a selection of 12 fish specimens sampled from Rapa Island. Results were obtained from one (rRBA and LC-MS/MS) and three independent experiments (CBA-N2a). For additional details, see Section 5.5.

Sampling Site	ID #	Scientific Name	Diet	rRBA	CBA-N2a *	LC-MS/MS
Iripau Bay	74	*Ctenochaetus striatus*	H	7.3	0.33 ± 0.05	<LOQ
Akaomua Bay	159	*Kyphosus cinerascens*	H	<LOD	<LOD	<LOQ
Motu Ta’una	143	*Kyphosus cinerascens*	H	1.4	0.52 ± 0.09	<LOQ
Akaomua Bay	161	*Chlorurus microrhinos*	H	<LOD	0.05 ± 0.01	<LOD
Motu Rapa Iti	209	*Chlorurus microrhinos*	H	7.8	0.31 ± 0.03	<LOQ
Akaomua Bay	163	*Leptoscarus vaigiensis*	H	<LOD	<LOD	<LOD
Motu Rapa Iti	214	*Leptoscarus vaigiensis*	H	5.0	4.75 ± 0.25	0.75
Akaomua Bay	167	*Epinephelus fasciatus*	C	<LOD	<LOD	<LOD
Tapuaki Bay	48	*Epinephelus fasciatus*	C	0.8	0.23 ± 0.02	<LOD
Ahurei Bay	229	*Epinephelus merra*	C	1.4	0.11 ± 0.02	<LOQ
Anatakuri Bay	198	*Monotaxis grandoculis*	C	<LOD	0.10 ± 0.01	<LOD
Motu Rapa Iti	211	*Pseudocaranx dentex*	C	1.0	1.18 ± 0.08	<LOQ

H: Herbivore. C: Carnivore. LOD: Limit of detection. LOQ: Limit of quantification. * CBA-N2a data correspond to the sum of the CTX-like activity measured in both LF90/10 and LF100 dry extracts.

**Table 5:** Results of the non-parametric Mann–Kendall test when applied to the average annual minimum (Tmin), maximum (Tmax), mean (Tm) values of atmospheric temperature, and sea surface temperature (SST) datasets available for Rapa between 1960 and 2016. This test was used to detect monotonic trends in the data series. The H0 hypothesis states that temperature data come from a population with independent measures and are identically distributed, whereas the HA hypothesis states temperature data follow a monotonic trend.

Temperature Data Series	Kendall’s Tau	2-Sided *p* Value	Alpha	Conclusion
Tmax	0.202 (2.14)	3.2 × 10^−2^	0.05	HA
Tmin	0.473 (5.07)	3.58 × 10^−7^	0.05	HA
Tmean	0.347 (3.71)	2.1 × 10^−4^	0.05	HA
SST (ERSSTv5)	0.067 (0.71)	0.48	0.05	H0

**Table 6:** Pearson correlation coefficient values (r, CI = 0.95) between Group for High Resolution Sea Surface Temperature (GHRSST) Level 4 MW_OI and Extended Reconstructed Sea Surface Temperature (ERSST)v5 sea surface temperature data series, and the Tmax, Tmin, and Tmean of atmospheric temperatures data series obtained from the Rapa synoptic weather station between 1998 and 2016.

Time Period (Scale)	GHRSST/ERSST	GHRSST/Tmax	GHRSST/Tmin	GHRSST/Tmean
1998–2016 (month)	0.91	0.95	0.92	0.94
1998–2016 (year)	0.25	0.86	0.78	0.87

## Supplementary Materials

The following are available online at https://www.mdpi.com/2072-6651/12/12/759/s1, Table S1: Toxicity data of Rapa fish, according to species and sampling sites, as assessed by rRBA, Table S2: Selected *m/z* transitions and LC-MS/MS instrument parameters used for the scheduled MRM method, Figure S1: Composite toxicity dose-response curves of N2a cells in OV- (open symbols) and OV+ (solid symbols) conditions when exposed to increasing concentrations of LF100 fish extracts and pure Pacific CTXs, Figure S2: Standardized declaration form for Ciguatera and seafood poisoning cases.
