# Supplementary material for: Evidence for the Range Expansion of Ciguatera in French Polynesia: A Revisit of the 2009 Mass-Poisoning Outbreak in Rapa Island (Australes Archipelago)
Source: Toxins (Basel). 2020 Dec 1;12(12):759. doi: 10.3390/toxins12120759 (PMC7759781; doi:10.3390/toxins12120759)
Supplement: Supplementary file 1 [file toxins-12-00759-s001.pdf]

Supplementary Materials: Evidence for the Range Expansion of Ciguatera in French Polynesia: A Revisit of the 2009 Mass-Poisoning Outbreak in Rapa Island (Australes Archipelago)

Mireille Chinain, Clémence Mahana iti Gatti, André Ung, Philippe Cruchet, Taina Revel, Jérôme Viallon, Manoëlla Sibat, Patrick Varney, Victoire Laurent, Philipp Hess and Hélène Taiana Darius

Table S1. Toxicity data of Rapa fish, according to species and sampling sites, as assessed by rRBA. Data are expressed in µg CTX3C eq kg $^{-1}$. Mean IC_{50} values for CTX3C and CTX1B were 0.62 and 0.26 ng mL $^{-1}$, respectively. Hence, a conversion factor of x0.42 should be applied to obtain rRBA CTX-like activity expressed in µg CTX1B eq kg $^{-1}$.

ID #	Size (cm)	Weight (g)	Feeding type [1]	Scientific name	Common name	Local name	rRBA
				**Akatamiro Bay and Motu Aturapa**			
01	33	656	1	*Chlorurus microrhinos*	Steephead Parrotfish	uhu raepuu	3.3
02	26	230	3	*Pseudocaranx dentex*	Thicklipped Jack	matu	4.5
03	31	590	1	*Kyphosus cinerascens*	Highfin Chub	karamami	3.5
04	28	460	1	*Kyphosus cinerascens*	Highfin Chub	karamami	2.4
05	30	545	1	*Kyphosus cinerascens*	Highfin Chub	karamami	4.1
06	27	410	1	*Kyphosus cinerascens*	Highfin Chub	karamami	3.4
07	27	255	3	*Priacanthus hamrur*	Crescent-tail Bigeye	mata ana ana	2.7
08	30	420	1	*Leptoscarus vaigiensis*	Seagrass Parrotfish	komokomo	4.5
09	34	535	1	*Leptoscarus vaigiensis*	Seagrass Parrotfish	komokomo	5.6
10	34	440	1	*Leptoscarus vaigiensis*	Seagrass Parrotfish	komokomo	2.2
11	35	740	3	*Parupeneus ciliatus*	Whitelined Goatfish	katuri	3.3
12	30	475	3	*Parupeneus ciliatus*	Whitelined Goatfish	katuri	5.4
13	31	500	3	*Parupeneus ciliatus*	Whitelined Goatfish	katuri	0.9
14	35	810	1	*Chlorurus microrhinos*	Steephead Parrotfish	uhu raepuu	3.8
15	36	950	3	*Caranx ignobilis*	Giant Jack	pa'aihere	3.7
16	32	555	1	*Leptoscarus vaigiensis*	Seagrass Parrotfish	komokomo	1.5
17	33	540	1	*Leptoscarus vaigiensis*	Seagrass Parrotfish	komokomo	3.8
18	25	360	1	*Kyphosus cinerascens*	Highfin Chub	karamami	1.4
19	25	340	1	*Kyphosus cinerascens*	Highfin Chub	karamami	1.0
				**Turoa Pari Ati Bay**			
20	25	278	3	*Epinephelus fasciatus*	Blacktip Grouper	rari	4.0
21	22	237	1	*Scarus psittacus*	Palenose Parrotfish	pahoro	4.3
22	26	286	3	*Goniistius plessisi*	Plessis' Morwong	pakakea	8.4
23	26	353	3	*Parupeneus ciliatus*	Whitelined Goatfish	katuri	5.8
24	28	340	3	*Pseudocaranx dentex*	Thicklipped Jack	matu	4.3
25	22	327	1	*Acanthurus leucopareius*	Whitebar Surgeonfish	maamaa	3.3
26	22	275	1	*Acanthurus leucopareius*	Whitebar Surgeonfish	maamaa	3.7
27	33	537	1	*Leptoscarus vaigiensis*	Seagrass Parrotfish	komokomo	4.5

28	33	548	1	*Leptoscarus vaigiensis*	Seagrass Parrotfish	komokomo	5.3
29	32	582	1	*Leptoscarus vaigiensis*	Seagrass Parrotfish	komokomo	4.1
30	35	789	1	*Leptoscarus vaigiensis*	Seagrass Parrotfish	komokomo	4.5
31	25	339	1	*Kyphosus cinerascens*	Highfin Chub	karamami	1.5
32	26	445	1	*Kyphosus cinerascens*	Highfin Chub	karamami	0.7
33	32	887	1	*Kyphosus cinerascens*	Highfin Chub	nanue	1.2
34	36	813	3	*Coris aygula*	Clown Coris	haupa	<LOD [2]
**Tapuaki Bay**							
35	19	183	1	*Acanthurus leucopareius*	Whitebar Surgeonfish	maamaa	1,3
36	23	278	1	*Acanthurus leucopareius*	Whitebar Surgeonfish	maamaa	2,8
37	28	315	3	*Pseudocaranx dentex*	Thicklipped Jack	matu	4,9
38	33	600	3	*Caranx ignobilis*	Giant Jack	pa'aihere	2,1
39	27	385	3	*Goniistius plessisi*	Plessis' Morwong	pakakea	8,6
40	26	290	3	*Parupeneus multifasciatus*	Manybar Goatfish	atiatia	1,3
41	30	593	3	*Parupeneus multifasciatus*	Manybar Goatfish	atiatia	2,7
42	23	246	1	*Kyphosus cinerascens*	Highfin Chub	karamami	0,9
43	27	442	1	*Kyphosus cinerascens*	Highfin Chub	karamami	1,8
44	27	325	1	*Leptoscarus vaigiensis*	Seagrass Parrotfish	komokomo	4,4
45	27	382	1	*Leptoscarus vaigiensis*	Seagrass Parrotfish	komokomo	4,4
46	29	430	1	*Leptoscarus vaigiensis*	Seagrass Parrotfish	komokomo	3,3
47	23	213	3	*Epinephelus fasciatus*	Blacktip Grouper	rari	<LOD
48	37	980	3	*Epinephelus fasciatus*	Blacktip Grouper	rari	0.8
49	44	1180	3	*Plectorhinchus picus*	Spotted Sweetlips	matuatua	2.7
50	33	746	1	*Chlorurus microrhinos*	Steephead Parrotfish	uhu raepuu	1.7
51	38	1110	1	*Chlorurus microrhinos*	Steephead Parrotfish	uhu raepuu	0.7
**Angananue Bay**							
52	35	630	1	*Leptoscarus vaigiensis*	Seagrass Parrotfish	komokomo	4.2
53	30	412	1	*Leptoscarus vaigiensis*	Seagrass Parrotfish	komokomo	2.9
54	26	315	1	*Kyphosus cinerascens*	Highfin Chub	karamami	0.7
55	26	370	1	*Kyphosus cinerascens*	Highfin Chub	karamami	0.8
56	23	235	1	*Kyphosus cinerascens*	Highfin Chub	karamami	0.9
57	23	315	1	*Acanthurus leucopareius*	Whitebar Surgeonfish	maamaa	0.7
58	20	210	1	*Acanthurus leucopareius*	Whitebar Surgeonfish	maamaa	1.0
59	25	247	3	*Priacanthus hamrur*	Crescent-tail Bigeye	mata ana ana	<LOD
60	24	175	3	*Priacanthus hamrur*	Crescent-tail Bigeye	mata ana ana	1.0
**Iripau Bay**							
61	39	1050	1	*Chlorurus microrhinos*	Steephead Parrotfish	uhu raepuu	<LOD
62	42	1190	1	*Chlorurus microrhinos*	Steephead Parrotfish	uhu raepuu	1.0
63	42	1315	1	*Kyphosus vaigiensis*	Lowfin Chub	karamami ume	2.2
64	31	535	1	*Kyphosus cinerascens*	Highfin Chub	karamami	1.1
65	36	690	3	*Parupeneus ciliatus*	Manybar Goatfish	katuri	1.3
66	36	985	3	*Parupeneus ciliatus*	Manybar Goatfish	katuri	1.1
67	35	620	1	*Leptoscarus vaigiensis*	Seagrass Parrotfish	komokomo	1.6

68	36	695	1	*Leptoscarus vaigiensis*	Seagrass Parrotfish	komokomo	1.4
69	29	335	3	*Lutjanus kasmira*	Bluelined Snapper	ta'ape	1.6
70	28	405	3	*Goniistius plessisi*	Plessis' Morwong	pakakea	4.8
71	24	280	1	*Acanthurus leucopareius*	Whitebar Surgeonfish	maamaa	1.5
72	22	255	1	*Acanthurus leucopareius*	Whitebar Surgeonfish	maamaa	1.5
73	23	310	1	*Ctenochaetus striatus*	Striated Surgeonfish	maito	1.0
74	22	250	1	*Ctenochaetus striatus*	Striated Surgeonfish	maito	7.3
**Ana Rua Bay**							
75	48	1700	3	*Plectorhinchus picus*	Spotted Sweetlips	matuatua	1.1
76	40	1170	1	*Chlorurus microrhinos*	Steephead Parrotfish	uhu raepuu	<LOD
77	30	465	1	*Chlorurus microrhinos*	Steephead Parrotfish	uhu raepuu	<LOD
78	30	400	3	*Parupeneus ciliatus*	Manybar Goatfish	katuri	1.4
79	35	690	1	*Leptoscarus vaigiensis*	Seagrass Parrotfish	komokomo	1.5
80	29	325	1	*Leptoscarus vaigiensis*	Seagrass Parrotfish	komokomo	1.2
81	42	1590	1	*Kyphosus vaigiensis*	Lowfin Chub	karamami ume	2.4
82	35	765	1	*Kyphosus cinerascens*	Highfin Chub	karamami	0.8
83	29	420	1	*Kyphosus cinerascens*	Highfin Chub	karamami	<LOD
84	23	280	1	*Acanthurus leucopareius*	Whitebar Surgeonfish	maamaa	<LOD
85	19	170	1	*Acanthurus leucopareius*	Whitebar Surgeonfish	maamaa	<LOD
86	22	310	1	*Ctenochaetus striatus*	Striated Surgeonfish	maito	<LOD
87	23	280	1	*Ctenochaetus striatus*	Striated Surgeonfish	maito	<LOD
**Agaira'o Bay**							
88	42	1425	1	*Chlorurus microrhinos*	Steephead Parrotfish	uhu raepuu	<LOD
89	35	775	1	*Chlorurus microrhinos*	Steephead Parrotfish	uhu raepuu	0.2
90	28	345	1	*Leptoscarus vaigiensis*	Seagrass Parrotfish	komokomo	2.2
91	25	290	1	*Leptoscarus vaigiensis*	Seagrass Parrotfish	komokomo	2.0
92	44	1370	3	*Plectorhinchus picus*	Spotted Sweetlips	matuatua	0.3
93	38	675	3	*Plectorhinchus picus*	Spotted Sweetlips	matuatua	0.2
94	37	746	1	*Leptoscarus vaigiensis*	Seagrass Parrotfish	komokomo	0.6
95	35	610	1	*Leptoscarus vaigiensis*	Seagrass Parrotfish	komokomo	0.6
96	38	990	1	*Chlorurus microrhinos*	Steephead Parrotfish	uhu raepuu	1.6
97	31	550	1	*Chlorurus microrhinos*	Steephead Parrotfish	uhu raepuu	<LOD
98	30	553	1	*Kyphosus cinerascens*	Highfin Chub	karamami	1.4
99	30	530	1	*Kyphosus cinerascens*	Highfin Chub	karamami	1.6
100	28	510	1	*Kyphosus cinerascens*	Highfin Chub	karamami	1.4
101	29	335	3	*Goniistius plessisi*	Plessis' Morwong	pakakea	10.0
102	29	430	3	*Parupeneus ciliatus*	Manybar Goatfish	katuri	2.0
103	19	192	1	*Ctenochaetus striatus*	Striated Surgeonfish	maito	6.0
104	18	170	1	*Acanthurus leucopareius*	Whitebar Surgeonfish	maamaa	1.0
**Piriauta Bay**							
105	50	1720	3	*Plectorhinchus picus*	Spotted Sweetlips	matuatua	1.3
106	44	1625	1	*Chlorurus microrhinos*	Steephead Parrotfish	uhu raepuu	0.7
107	40	1280	1	*Chlorurus microrhinos*	Steephead Parrotfish	uhu raepuu	2.5

108	64	2740	3	*Seriola lalandi*	King Fish	ma'aki	0.9
109	50	2160	1	*Chlorurus microrhinos*	Steephead Parrotfish	uhu raepuu	1.4
110	45	1580	1	*Chlorurus microrhinos*	Steephead Parrotfish	uhu raepuu	2.1
111	37	1340	1	*Kyphosus cinerascens*	Highfin Chub	nanue	0.9
112	36	1060	1	*Kyphosus cinerascens*	Highfin Chub	nanue	2.2
113	36	670	3	*Parupeneus ciliatus*	Manybar Goatfish	katuri	1.8
114	35	705	3	*Parupeneus ciliatus*	Manybar Goatfish	katuri	1.8
115	33	765	1	*Kyphosus cinerascens*	Highfin Chub	karamami	1.4
116	31	714	1	*Kyphosus cinerascens*	Highfin Chub	karamami	1.5
117	37	750	3	*Epinephelus fasciatus*	Blacktip Grouper	rari	<LOD
118	34	565	3	*Epinephelus fasciatus*	Blacktip Grouper	rari	<LOD
119	36	690	1	*Leptoscarus vaigiensis*	Seagrass Parrotfish	komokomo	3.5
120	33	545	1	*Leptoscarus vaigiensis*	Seagrass Parrotfish	komokomo	1.7
121	23	300	1	*Acanthurus leucopareius*	Whitebar Surgeonfish	maamaa	<LOD
122	21	235	1	*Acanthurus leucopareius*	Whitebar Surgeonfish	maamaa	1.3
123	23	269	3	*Myripristis* sp.	Soldierfish	i'ihi	2.7
124	22	266	3	*Myripristis* sp.	Soldierfish	i'ihi	2.7
				**Motu Tarakoi**			
125	56	2540	3	*Epinephelus tauvina*	Greasy Grouper	faroa	<LOD
126	44	1480	1	*Chlorurus microrhinos*	Steephead Parrotfish	uhu raepuu	1.2
127	34	800	1	*Chlorurus microrhinos*	Steephead Parrotfish	uhu raepuu	1.5
128	37	760	1	*Leptoscarus vaigiensis*	Seagrass Parrotfish	komokomo	1.5
129	36	680	1	*Leptoscarus vaigiensis*	Seagrass Parrotfish	komokomo	1.6
130	42	1290	3	*Plectorhinchus picus*	Spotted Sweetlips	matuatua	0.8
131	40	980	3	*Plectorhinchus picus*	Spotted Sweetlips	matuatua	0.6
132	37	1250	1	*Kyphosus cinerascens*	Highfin Chub	nanue	1.2
133	36	1160	1	*Kyphosus cinerascens*	Highfin Chub	nanue	2.0
134	24	270	1	*Ctenochaetus striatus*	Striated Surgeonfish	maito	1.7
135	23	270	1	*Ctenochaetus striatus*	Striated Surgeonfish	maito	4.2
136	33	810	1	*Kyphosus cinerascens*	Highfin Chub	karamami	0.6
137	30	540	1	*Kyphosus cinerascens*	Highfin Chub	karamami	0.9
138	25	305	3	*Myripristis* sp.	Soldierfish	i'ihi	4.0
139	25	430	1	*Acanthurus leucopareius*	Whitebar Surgeonfish	maamaa	0.8
140	20	190	1	*Acanthurus leucopareius*	Whitebar Surgeonfish	maamaa	1.3
				**Motu Ta'una**			
141	72	7750	1	*Kyphosus vaigiensis*	Lowfin Chub	ume	1.0
142	63	5150	1	*Kyphosus vaigiensis*	Lowfin Chub	ume	0.5
143	38	1365	1	*Kyphosus cinerascens*	Highfin Chub	nanue	1.4
144	36	970	1	*Kyphosus cinerascens*	Highfin Chub	nanue	1.0
145	45	1430	3	*Plectorhinchus picus*	Spotted Sweetlips	matuatua	1.4
146	44	1670	1	*Chlorurus frontalis*	Tan-faced Parrotfish	uhu naonao	<LOD
147	38	970	1	*Chlorurus microrhinos*	Steephead Parrotfish	uhu raepuu	0.7
148	32	645	1	*Chlorurus microrhinos*	Steephead Parrotfish	uhu raepuu royal	1.3

149	34	856	1	*Kyphosus cinerascens*	Highfin Chub	karamami	<LOD
150	31	575	1	*Kyphosus cinerascens*	Highfin Chub	karamami	<LOD
151	37	1070	1	*Leptoscarus vaigiensis*	Seagrass Parrotfish	komokomo	1.5
152	35	690	1	*Leptoscarus vaigiensis*	Seagrass Parrotfish	komokomo	0.7
153	37	780	3	*Epinephelus fasciatus*	Blacktip Grouper	rari	0.8
154	26	425	3	*Myripristis* sp.	Soldierfish	i'ihi	1.9
155	24	320	3	*Myripristis* sp.	Soldierfish	i'ihi	1.3
156	26	310	3	*Goniistius plessisi*	Plessis' Morwong	pakakea	14.5
157	22	300	1	*Acanthurus leucopareius*	Whitebar Surgeonfish	maamaa	1.5
158	20	215	1	*Acanthurus leucopareius*	Whitebar Surgeonfish	maamaa	<LOD
**Akaomua Bay**							
159	56	3450	1	*Kyphosus cinerascens*	Highfin Chub	pakavai	<LOD
160	52	3015	1	*Kyphosus cinerascens*	Highfin Chub	pakavai	<LOD
161	41	1290	1	*Chlorurus microrhinos*	Steephead Parrotfish	uhu raepuu	<LOD
162	36	760	1	*Chlorurus microrhinos*	Steephead Parrotfish	uhu raepuu	<LOD
163	37	785	1	*Leptoscarus vaigiensis*	Seagrass Parrotfish	komokomo	<LOD
164	34	590	1	*Leptoscarus vaigiensis*	Seagrass Parrotfish	komokomo	<LOD
165	34	635	3	*Parupeneus ciliatus*	Manybar Goatfish	katuri	<LOD
166	28	570	3	*Parupeneus ciliatus*	Manybar Goatfish	katuri	0.8
167	34	530	3	*Epinephelus fasciatus*	Blacktip Grouper	rari	<LOD
168	29	355	3	*Epinephelus fasciatus*	Blacktip Grouper	rari	<LOD
169	29	530	1	*Kyphosus cinerascens*	Highfin Chub	karamami	<LOD
170	24	310	1	*Kyphosus cinerascens*	Highfin Chub	karamami	<LOD
171	19	235	1	*Acanthurus leucopareius*	Whitebar Surgeonfish	maamaa	<LOD
172	18	180	1	*Acanthurus leucopareius*	Whitebar Surgeonfish	maamaa	<LOD
173	25	200	3	*Priacanthus hamrur*	Crescent-tail Bigeye	mata ana ana	<LOD
**Makatea Peak**							
174	66	2300	3	*Pseudocaranx dentex*	Thicklipped Jack	matu	<LOD
175	37	730	3	*Pseudocaranx dentex*	Thicklipped Jack	matu	<LOD
176	48	2010	1	*Scarus altipinnis*	Filament-finned Parrotfish	haumeretue	<LOD
177	46	1460	1	*Scarus altipinnis*	Filament-finned Parrotfish	roro	<LOD
**Anatakuri Bay**							
178	39	160	1	*Kyphosus cinerascens*	Highfin Chub	nanue	1.2
179	36	1280	1	*Kyphosus cinerascens*	Highfin Chub	nanue	0.7
180	44	1240	3	*Plectorhinchus picus*	Spotted Sweetlips	matuatua	<LOD
181	36	700	3	*Plectorhinchus picus*	Spotted Sweetlips	matuatua	0.5
182	42	1345	1	*Chlorurus microrhinos*	Steephead Parrotfish	uhu raepuu	1.0
183	42	1250	1	*Chlorurus microrhinos*	Steephead Parrotfish	uhu raepuu	<LOD
184	35	870	1	*Kyphosus cinerascens*	Highfin Chub	karamami	<LOD
185	33	800	1	*Kyphosus cinerascens*	Highfin Chub	karamami	0.5
186	37	720	1	*Leptoscarus vaigiensis*	Seagrass Parrotfish	komokomo	0.5
187	33	615	1	*Leptoscarus vaigiensis*	Seagrass Parrotfish	komokomo	1.9

188	33	450	3	*Epinephelus fasciatus*	Blacktip Grouper	rari	<LOD
189	61	2300	1	*Chlorurus microrhinos*	Steephead Parrotfish	uhu raepuu	1.3
190	42	1460	1	*Chlorurus microrhinos*	Steephead Parrotfish	uhu raepuu royal	1.1
191	47	1540	3	*Pseudocaranx dentex*	Thicklipped Jack	matu	<LOD
192	38	800	3	*Pseudocaranx dentex*	Thicklipped Jack	matu	1.0
193	44	1530	3	*Plectorhinchus picus*	Spotted Sweetlips	matuatua	0.6
194	35	1080	1	*Kyphosus cinerascens*	Highfin Chub	nanue	0.5
195	34	810	1	*Kyphosus cinerascens*	Highfin Chub	karamami	0.7
196	32	465	3	*Goniistius plessisi*	Plessis' Morwong	pakakea	6.3
197	29	375	3	*Goniistius plessisi*	Plessis' Morwong	pakakea	6.3
198	37	1250	3	*Monotaxis grandoculis*	Bigeye Emperor	mu	<LOD
199	35	545	1	*Leptoscarus vaigiensis*	Seagrass Parrotfish	komokomo	<LOD
200	35	575	1	*Leptoscarus vaigiensis*	Seagrass Parrotfish	komokomo	0.7
201	39	810	3	*Epinephelus fasciatus*	Blacktip Grouper	rari	0.6
202	34	535	3	*Epinephelus fasciatus*	Blacktip Grouper	rari	0.6
203	26	370	1	*Naso unicornis*	Brown Unicornfish	ume tara	2.9
204	20	268	1	*Acanthurus leucopareius*	Whitebar Surgeonfish	maamaa	1.5
205	20	230	1	*Acanthurus leucopareius*	Whitebar Surgeonfish	maamaa	2.6
**Motu Rapa Iti**							
206	69	7200	1	*Kyphosus vaigiensis*	Lowfin Chub	ume	5.7
207	52	3140	1	*Kyphosus cinerascens*	Highfin Chub	pakavai	1.2
208	47	2100	1	*Chlorurus microrhinos*	Steephead Parrotfish	uhu raepuu	9.4
209	43	1450	1	*Chlorurus microrhinos*	Steephead Parrotfish	uhu raepuu	7.8
210	48	1890	3	*Plectorhinchus picus*	Spotted Sweetlips	matuatua	<LOD
211	43	1320	3	*Pseudocaranx dentex*	Thicklipped Jack	matu	1.0
212	37	800	3	*Pseudocaranx dentex*	Thicklipped Jack	matu	1.4
213	37	1000	1	*Kyphosus cinerascens*	Highfin Chub	nanue	<LOD
214	39	910	1	*Leptoscarus vaigiensis*	Seagrass Parrotfish	komokomo	5.0
215	37	700	1	*Leptoscarus vaigiensis*	Seagrass Parrotfish	komokomo	2.0
216	28	490	1	*Kyphosus cinerascens*	Highfin Chub	karamami	0.6
217	30	485	1	*Kyphosus cinerascens*	Highfin Chub	karamami	<LOD
218	31	400	3	*Goniistius plessisi*	Plessis' Morwong	pakakea	8.5
219	29	360	3	*Goniistius plessisi*	Plessis' Morwong	pakakea	10.8
220	23	315	1	*Acanthurus leucopareius*	Whitebar Surgeonfish	maamaa	1.3
221	23	280	1	*Acanthurus leucopareius*	Whitebar Surgeonfish	maamaa	2.3
**Ahurei Bay**							
222	62	3680	3	*Plectropomus laevis*	Blacksaddle Coralgrouper	tonu	2.1
223	48	1875	3	*Plectorhinchus picus*	Spotted Sweetlips	matuatua	1.5
224	47	1605	3	*Pseudocaranx dentex*	Thicklipped Jack	matu	1.9
225	40	990	3	*Pseudocaranx dentex*	Thicklipped Jack	matu	2.4
226	31	615	1	*Kyphosus cinerascens*	Highfin Chub	karamami	2.5
227	31	570	1	*Kyphosus cinerascens*	Highfin Chub	karamami	2.0
228	27	240	3	*Epinephelus merra*	Honeycomb Grouper	tarao	<LOD

229	26	245	3	*Epinephelus merra*	Honeycomb Grouper	tarao	1.4
230	27	310	3	*Epinephelus merra*	Honeycomb Grouper	tarao	<LOD
231	28	300	3	*Epinephelus merra*	Honeycomb Grouper	tarao	<LOD
232	32	535	3	*Pseudocaranx dentex*	Thicklipped Jack	matu	1.1
233	25	270	3	*Pseudocaranx dentex*	Thicklipped Jack	matu	1.0
234	53	2610	3	*Caranx ignobilis*	Giant Jack	pa'aihere	1.7
235	50	2260	1	*Chlorurus microrhinos*	Steephead Parrotfish	uhu raepuu	10.7
236	42	1680	1	*Chlorurus microrhinos*	Steephead Parrotfish	uhu raepuu	8.8
237	34	680	1	*Leptoscarus vaigiensis*	Seagrass Parrotfish	komokomo	12.4
238	34	610	1	*Leptoscarus vaigiensis*	Seagrass Parrotfish	komokomo	2.7
239	27	415	1	*Kyphosus cinerascens*	Highfin Chub	karamami	1.2
240	26	345	1	*Kyphosus cinerascens*	Highfin Chub	karamami	1.8
241	32	470	3	*Goniistius plessisi*	Plessis' Morwong	pakakea	9.7
242	40	1175	3	*Caranx ignobilis*	Giant Jack	pa'aihere	0.6
243	29	430	3	*Pseudocaranx dentex*	Thicklipped Jack	matu	2.0
244	29	385	3	*Pseudocaranx dentex*	Thicklipped Jack	matu	1.6
245	34	420	2	*Crenimugil crenilabis*	Fringelip Mullet	tehu	1.5
246	27	330	3	*Priacanthus hamrur*	Crescent-tail Bigeye	mata ana ana	1.1
247	24	213	3	*Priacanthus hamrur*	Crescent-tail Bigeye	mata ana ana	1.4
248	27	385	3	*Lutjanus kasmira*	Bluelined Snapper	ta'ape	1.1
249	23	255	1	*Kyphosus cinerascens*	Highfin Chub	karamami	<LOD
250	40	856	2	*Crenimugil crenilabis*	Fringelip Mullet	tehu	<LOD
251	33	628	2	*Crenimugil crenilabis*	Fringelip Mullet	tehu	1.7

[1]Feeding type codes: (1) mainly plants/detritus - (2) plants/detritus + animals – (3) mainly animals.

[2]Fish samples with a CTX-like activity < 0.31 µg CTX3C eq kg $^{-1}$.

Table S2. Selected *m/z* transitions and LC-MS/MS instrument parameters used for the scheduled MRM method.

Compound	Detection window (min)	Precursor ion (Q1) *m/z*	Product ion (Q3) *m/z*	DP (eV)	CE (eV)	CXP (eV)
CTX1B	3.1 ± 1	1128.6 $[M+NH_4]^+$	1093.6	105	20	12
			1075.6	105	30	12
			95.1	105	90	20
M-*seco*-CTX3C	4.7 ± 1	1041.6 $[M+H]^+$	1023.6	105	30	12
			1005.6	105	20	12
			125.1	105	50	18
2-hydroxyCTX3C and 3-hydroxyCTX3C	5.4 ± 1	1058.6 $[M+NH_4]^+$	1023.6	105	30	12
			1005.6	105	20	12
			125.1	105	50	18
2,3-dihydroxyCTX3C	6.0 ± 1	1074.6 $[M+NH_4]^+$	1039.6	105	30	12
		1057.6 $[M+H]^+$	1039.6	105	20	12
			125.1	105	50	18
51-hydroxyCTX3C	6.3 ± 1	1056.6 $[M+NH_4]^+$	1021.6	105	30	12
		1039.6 $[M+H]^+$	1021.6	105	20	12
			1003.6	105	20	12
M-*seco*-CTX4A/B	6.5 ± 1	1096.6 $[M+NH_4]^+$	1043.7	105	30	12
		1079.6 $[M+H]^+$	1043.7	105	20	12
			125.1	105	50	18
52-*epi*-54-deoxyCTX1B and 54-deoxyCTX1B	6.8 ± 1	1112.6 $[M+NH_4]^+$	1077.6	105	20	12
			1059.6	105	30	12
			95.1	105	90	20
CTX3C isomers (1), (2) and (3)	7.6 ± 1	1040.6 $[M+NH_4]^+$	1005.6	105	30	12
		1023.6 $[M+H]^+$	1005.6	105	20	12
			125.1	105	20	12
CTX3C, CTX3B and CTX3C isomer (4)	10.5 ± 1	1040.6 $[M+NH_4]^+$	1005.6	105	30	12
		1023.6 $[M+H]^+$	1005.6	105	20	12
			125.1	105	50	18
CTX4A and CTX4B	12.2 ± 1	1078.6 $[M+NH_4]^+$	1043.6	105	30	12
		1061.6 $[M+H]^+$	1043.6	105	20	12
			125.1	105	50	18

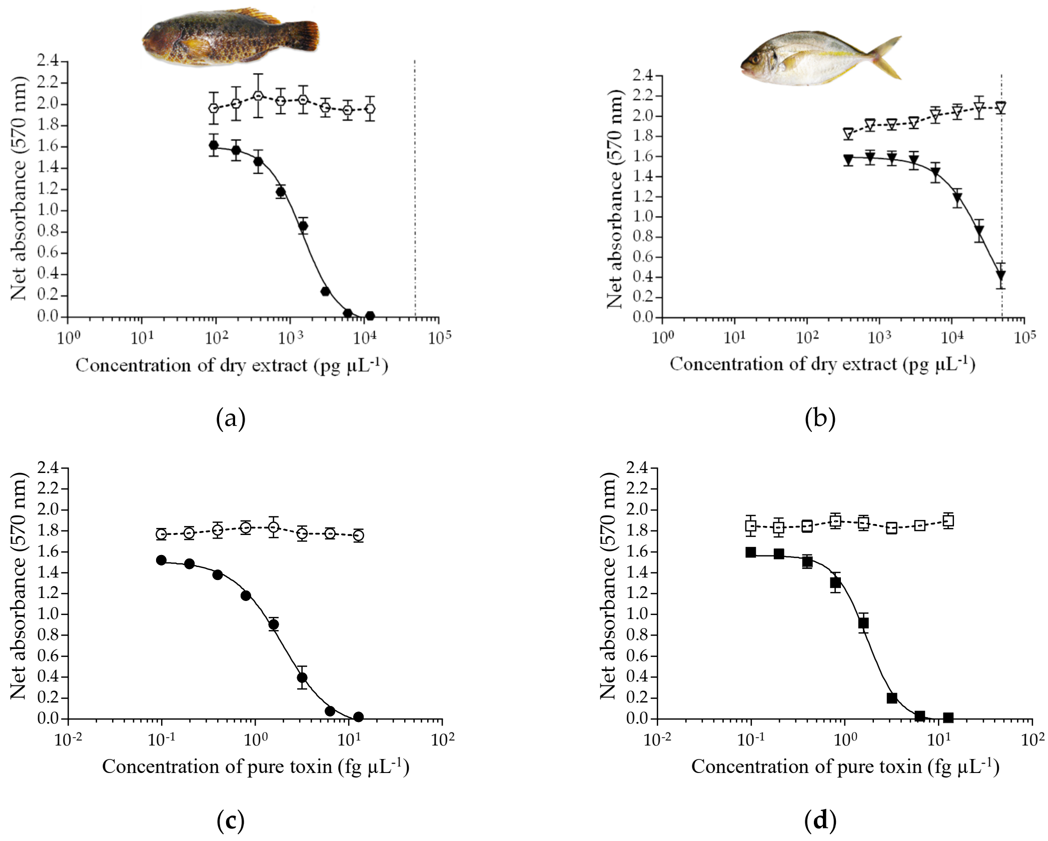

Figure S1. Composite toxicity dose-response curves of N2a cells in OV- (open symbols) and OV+ (solid symbols) conditions when exposed to increasing concentrations of LF100 extracts and pure Pacific CTXs. (**a**) *Leptoscarus vaigiensis* #214; (**b**) *Pseudocaranx dentex* #211; (**c**) CTX3C; (**d**) CTX1B. Data represent the mean ± SD of three independent experiments, each concentration run in triplicate (n = 9). The dotted vertical line corresponds to the maximum concentration of LF100 dry extracts for matrix interference (MCE = 50,000 pg µL $^{-1}$). Mean EC_{50} values for CTX3C and CTX1B were 1.91 ± 0.22 and 1.72 ± 0.17 fg µL $^{-1}$ (from three independent experiments).

Réseau de Surveillance de la Ciguatéra

RSC

ilm

DIRECTION DE LA SANTE BUREAU DE VEILLE SANITAIRE

French Polynesia Ciguatera and seafood poisoning Surveillance Network

DECLARATION FORM

PATIENT

Age ____ years Sex: ☐ F ☐ M

CONTEXT OF POISONING

Date of consumption ____

Local name of marine product responsible for the poisoning ____

Part(s) consumed ☐ Flesh ☐ Head ☐ Viscera ☐ Eggs

Specify the fishing spot (Mark with a cross on the map)

Region/town ____ Island ____

☐ bought on the roadside ☐ bought at the market / store (specify) ____

CLINICAL DATA

For information: if the patient presents with fever patient and / or allergic reactions and / or skin rash, the diagnosis of ciguatera must be rejected.

INTENSITY Mild	Moderate	High	
			**CARDIOVASCULAR SIGNS**
☐	☐	☐	Bradycardia
☐	☐	☐	Tachycardia
☐	☐	☐	Hypotension
☐	☐	☐	Hypertension
			**DIGESTIVE SIGNS**
☐	☐	☐	Nausea
☐	☐	☐	Vomiting
☐	☐	☐	Diarrhea
			**GENERAL AND NEUROLOGICAL SIGNS**
☐	☐	☐	Tingling extremities (hands, feet)
☐	☐	☐	Touch, neuro-sensitives disturbances
☐	☐	☐	Dysesthesia (in contact with cold / hot)
☐	☐	☐	Itching
☐	☐	☐	Asthenia
☐	☐	☐	Headache
☐	☐	☐	Dizziness / Balance or walking disorders (underline)
☐	☐	☐	Vision disorders
☐	☐	☐	Muscular disorders (pain, cramps, weaknesses ...)
☐	☐	☐	Joint pain
☐	☐	☐	Hypotermia : Temperature ____ °C
☐	☐	☐	Burn / tingling of lips, mouth, throat
☐	☐	☐	Orofacial pain (teeth, jaw, face)
☐	☐	☐	Dysgueusia (taste alteration)
☐	☐	☐	Urogenital discomfort/ itching / burning
☐	☐	☐	Hallucinations

Other symptoms/Observations ____

Time elapsed between the meal and the onset of symptoms ☐ < 30 min ☐ < 2h ☐ < 12h ☐ > 12h

Number of previous CFP/PSP ____

Number of other person(s) also concerned by the poisoning *(in addition to the patient)* ____

MEDICAL STRUCTURE IDENTIFICATION

Date of consultation ____ Island/ Town ____

Medical structure ☐ First aid post ☐ Infirmary ☐ Medical Center ☐ Hospital ☐ Private ☐ Other

Please send the form to

*LMT - Institut Louis Malardé BP 30 98713 TAHITI / Tél: (689) 40 416 411 - **Fax: (689) 40 416 406** / Mail: **veille.ciguatera@ilm.pf***

*Vous avez également la possibilité de faire la **déclaration directement en ligne sur www.ciguatera.pf***

Figure S2. Standardized declaration form for Ciguatera and seafood poisoning cases.
